# Effects of Benzofuran–Chalcone, Benzofuran–Pyrazoline, and Bis-Pyrazoline Derivatives on Redox-Related Biomarkers and Fatty Acid, Vitamin, and Sterol Profiles in *Saccharomyces cerevisiae*

**DOI:** 10.3390/molecules31203769

**Published:** 2026-10-20

**Authors:** Buket Erzen, Ökkeş Yılmaz, Demet Coşkun, Ahmet Harun Evren, Mukaddes Karataş

**Affiliations:** 1 Department of Chemical Engineering, Faculty of Engineering, Fırat University, 23200 Elazığ, Türkiye; bcetintas@firat.edu.tr; 2 Department of Biology, Faculty of Science, Fırat University, 23200 Elazığ, Türkiye; oyilmaz@firat.edu.tr (Ö.Y.); hevren@firat.edu.tr (A.H.E.); 3 Department of Chemistry, Faculty of Science, Fırat University, 23200 Elazığ, Türkiye; dcoskun@firat.edu.tr

**Keywords:** benzofuran, chalcone, pyrazoline, bis-pyrazoline, redox-related biomarkers, lipid peroxidation, fatty acid profile, lipophilic vitamins, *Saccharomyces cerevisiae*

## Abstract

Benzofuran-based hybrid compounds have reported radical-scavenging and biological activities, but their cellular biochemical response profiles require further characterization. This study evaluated previously synthesized benzofuran–chalcone, benzofuran–pyrazoline, and bis-pyrazoline derivatives by combining cell-free assays with redox-related and lipid-associated biochemical measurements in *Saccharomyces cerevisiae*. Chemical radical-scavenging activity was assessed using the 2,2-diphenyl-1-picrylhydrazyl (DPPH) assay, and in vitro lipid peroxidation-related responses were evaluated using the thiobarbituric acid-reactive substances (TBARS) assay. Cellular measurements included reduced glutathione (GSH), malondialdehyde (MDA), total protein, fatty acids, and lipophilic vitamins and sterols. Cellular experiments comprised four independent experimental sets, with statistical comparisons restricted to each treatment and its corresponding concurrently prepared control. Compounds **1d** and **2b** showed comparatively high DPPH radical-scavenging responses under the tested conditions, whereas the in vitro TBARS responses varied with compound and treatment level. Cellular GSH and MDA changes were not consistently parallel: **2e** increased both parameters, **2f** markedly decreased GSH without significantly altering MDA, and **1b** and **2b** decreased GSH while increasing MDA. Treatments also altered fatty acid, tocopherol, and sterol profiles relative to their respective controls. These findings show that chemical radical-scavenging responses did not consistently correspond to cellular redox-related responses and that lipid-associated measurements provided complementary biochemical information. The study therefore characterizes distinct treatment-associated response profiles rather than a uniform antioxidant or pro-oxidant effect. Unequal molar exposures and the absence of concurrent viability and mechanistic measurements preclude definitive potency rankings, structure–activity relationships, or mechanistic conclusions.

## 1. Introduction

Oxidative stress arises from an imbalance between the production of reactive oxygen species (ROS) and the capacity of cellular antioxidant defense systems to counteract their effects. Persistent redox imbalance has been associated with the development and progression of numerous chronic and degenerative disorders, including neurodegenerative diseases, cancer, cardiovascular diseases, and metabolic disorders [1,2,3]. At the cellular level, excessive ROS generation, including superoxide anions, hydroxyl radicals, and hydrogen peroxide, can promote oxidative damage to lipids, proteins, and nucleic acids, thereby affecting membrane integrity, enzymatic function, and genomic stability. To counteract these effects, cells rely on an integrated antioxidant network comprising enzymatic defenses, including superoxide dismutase (SOD), catalase (CAT), and glutathione peroxidase, together with non-enzymatic antioxidant systems in which GSH plays an important role [4,5].

Redox homeostasis is closely interconnected with cellular lipid composition and membrane-associated processes. Lipophilic antioxidants, particularly tocopherols, can contribute to the protection of cellular lipids against oxidative damage, whereas fatty acid composition and sterol content are important determinants of membrane organization and function [6]. Under conditions of sustained oxidative stress, enhanced lipid peroxidation (LPO) may be accompanied by changes in cellular lipid composition and broader biochemical homeostasis. Accordingly, considerable research has focused on compounds with chemical radical-scavenging properties and on their associations with cellular redox-related responses and lipid-associated biochemical profiles [7,8].

Benzofuran-based scaffolds have attracted interest because their derivatives exhibit diverse biological activities, including antioxidant, antimicrobial, anti-inflammatory, anticancer, and neuroprotective effects [9,10]. The benzofuran core can be incorporated into hybrid structures containing chalcone or pyrazoline motifs, both of which have reported redox-related and other biological activities [11,12,13]. These hybrid frameworks provide a basis for investigating how structurally diverse derivatives behave in chemical assays and cellular systems [14,15]. The present study evaluated benzofuran–chalcone and benzofuran–pyrazoline derivatives together with one structurally distinct bis-pyrazoline derivative.

The positions and types of substituents on benzofuran-containing scaffolds have been associated with differences in biological activity, supporting the investigation of structurally diversified derivatives [16]. In chalcones, the α,β-unsaturated carbonyl system and variations in substituent type, position, and electronic characteristics have been associated with differences in chemical radical-scavenging behavior [17]. Similarly, pyrazolines are nitrogen-containing five-membered heterocycles whose biological activities can vary according to structural modification [18]. These observations support examining both chemical radical-scavenging behavior and cellular biochemical responses when evaluating such derivatives.

The synthetic versatility and biological applications of chalcones have been reviewed by Shalaby et al. (2023) [19]. Recent work by Al-Qadhi et al. (2026) extended this structural diversification to khellin-derived benzofuran–pyrazoline and benzofuran–pyrazoline–thiazolidinone hybrids investigated as anticancer agents [20]. In a related, non-benzofuran series, Hussaini et al. (2016) evaluated triazole-linked chalcone and pyrazoline derivatives and reported that selected compounds induced G2/M cell-cycle accumulation, inhibited tubulin polymerization, and activated apoptosis-associated caspases [21]. These studies illustrate the biological potential of such hybrid frameworks while emphasizing that their effects must be characterized within defined experimental models.

Ali et al. (2023) evaluated substituted benzofuran-linked chalcones using α-amylase inhibition and DPPH and ABTS radical-scavenging assays, reporting enzyme-inhibitory activity together with radical-scavenging potential [22]. Coşkun et al. (2017) investigated benzofuran-substituted chalcones in breast, lung, and prostate cancer cell lines and found that a selected derivative produced cytotoxic effects and induced caspase-dependent apoptosis [23]. Hassan et al. (2014) examined benzofuran–morpholinomethyl–pyrazoline hybrids in isolated rat thoracic aortic rings and reported vasorelaxant activity, with several compounds showing greater activity than the reference agent under their experimental conditions [24]. These investigations establish biological activity in specific assay systems, but their findings do not define the redox-related and lipid-associated biochemical responses to the derivatives evaluated in the present study.

A complementary question is how exposure to these derivatives is associated with cellular GSH and MDA levels alongside fatty acid, lipophilic vitamin, and sterol profiles. Assessing these endpoints together can broaden biochemical characterization beyond cell-free radical-scavenging measurements by considering redox-related responses in the context of cellular lipid composition [25,26,27]. This approach provides the rationale for examining chemical assay responses and cellular biochemical profiles within the same investigation.

*S. cerevisiae* provides a well-established eukaryotic model for investigating cellular redox biology and lipid-associated processes. Reviews of yeast glycerolipid regulation and systems biology describe its value for studying interconnected lipid, protein, and energy metabolism [28,29]. In an experimental study, Howlett and Avery (1997) enriched *S. cerevisiae* with polyunsaturated fatty acids and examined its responses to copper and cadmium exposure [30]. They found that increased fatty acid unsaturation was associated with greater metal sensitivity and lipid peroxidation, illustrating the relevance of lipid composition when interpreting cellular oxidative responses [30]. These findings support including fatty acid profiling alongside redox-related biochemical measurements. Nevertheless, observations in yeast require validation in appropriate mammalian systems before their broader biological relevance can be established [31].

The present study aimed to characterize the chemical radical-scavenging behavior and cellular biochemical responses of previously reported benzofuran–chalcone, benzofuran–pyrazoline, and bis-pyrazoline derivatives. DPPH radical-scavenging and in vitro TBARS assays were combined with measurements of intracellular GSH and MDA, total protein, fatty acid composition, and lipophilic vitamin and sterol profiles in *S. cerevisiae*. The contribution of this work lies in assessing these complementary endpoints within the same investigation to examine whether chemical radical-scavenging responses are accompanied by consistent cellular redox-related changes and to characterize the associated lipid profiles. Cellular findings were interpreted relative to the concurrently prepared control of each of four independent experimental sets, without cross-set statistical comparisons or definitive structure–activity claims.

## 2. Results

Cellular results are presented by experimental set, each with its own concurrently prepared vehicle control. Statistical comparisons were restricted to treatments versus their corresponding control within each set; no cross-set statistical comparisons were performed.

### 2.1. Benzofuran Derivatives Evaluated in the Present Study

**Figure 1:**
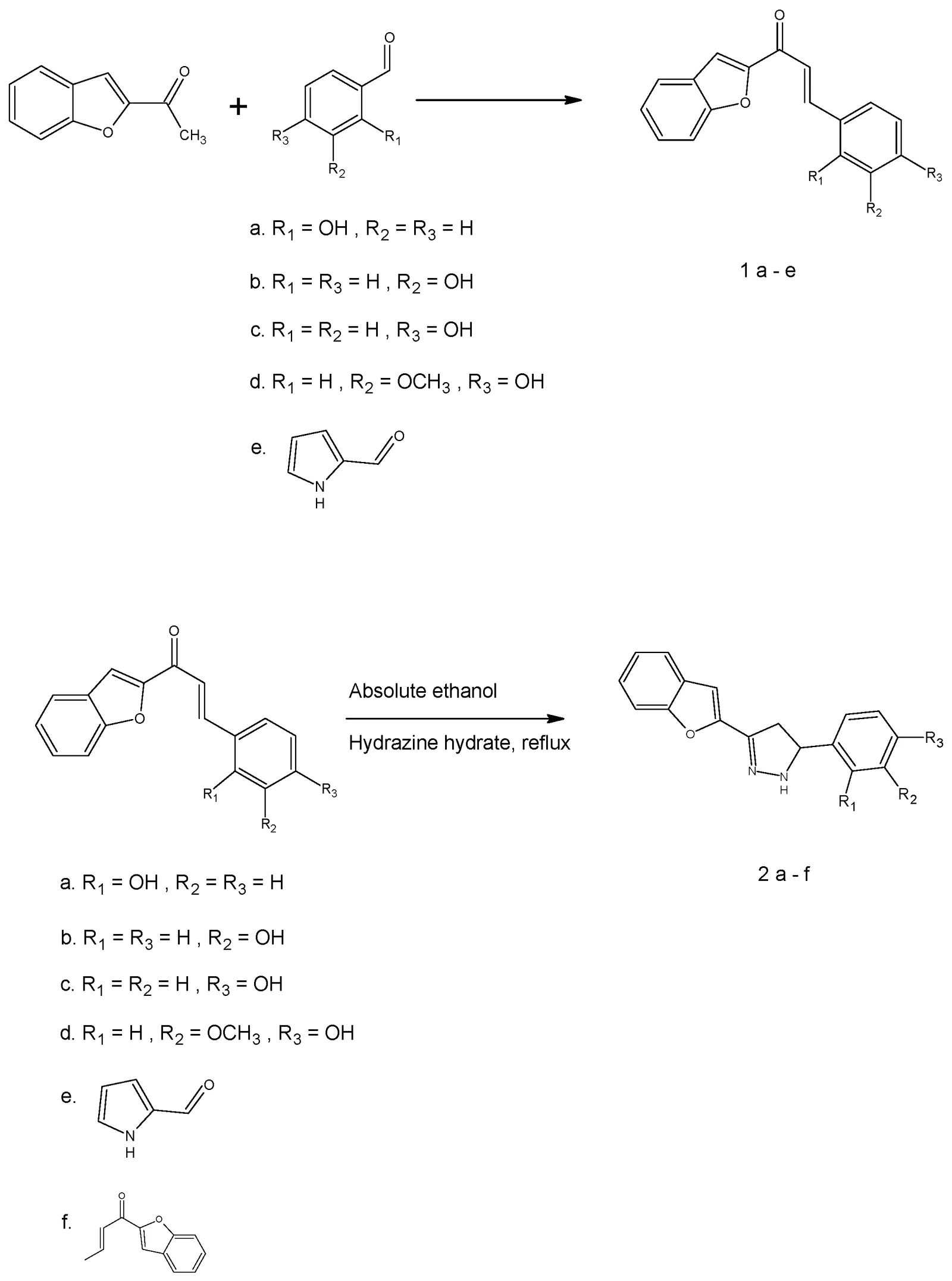
Chemical structures of the previously reported benzofuran–chalcone derivatives (**1a**–**1e**), benzofuran–pyrazoline derivatives (**2a**–**2e**), and the bis-pyrazoline derivative (**2f**) evaluated in the present study.

The evaluated compounds comprised five benzofuran–chalcone derivatives (**1a**–**1e**), five benzofuran–pyrazoline derivatives (**2a**–**2e**), and one bis-pyrazoline derivative (**2f**). Their synthesis and spectroscopic characterization were previously reported by Coşkun et al. [32,33], and their structures are shown in Figure 1.

### 2.2. DPPH Radical-Scavenging Activity of the Tested Compounds

**Figure 2:**
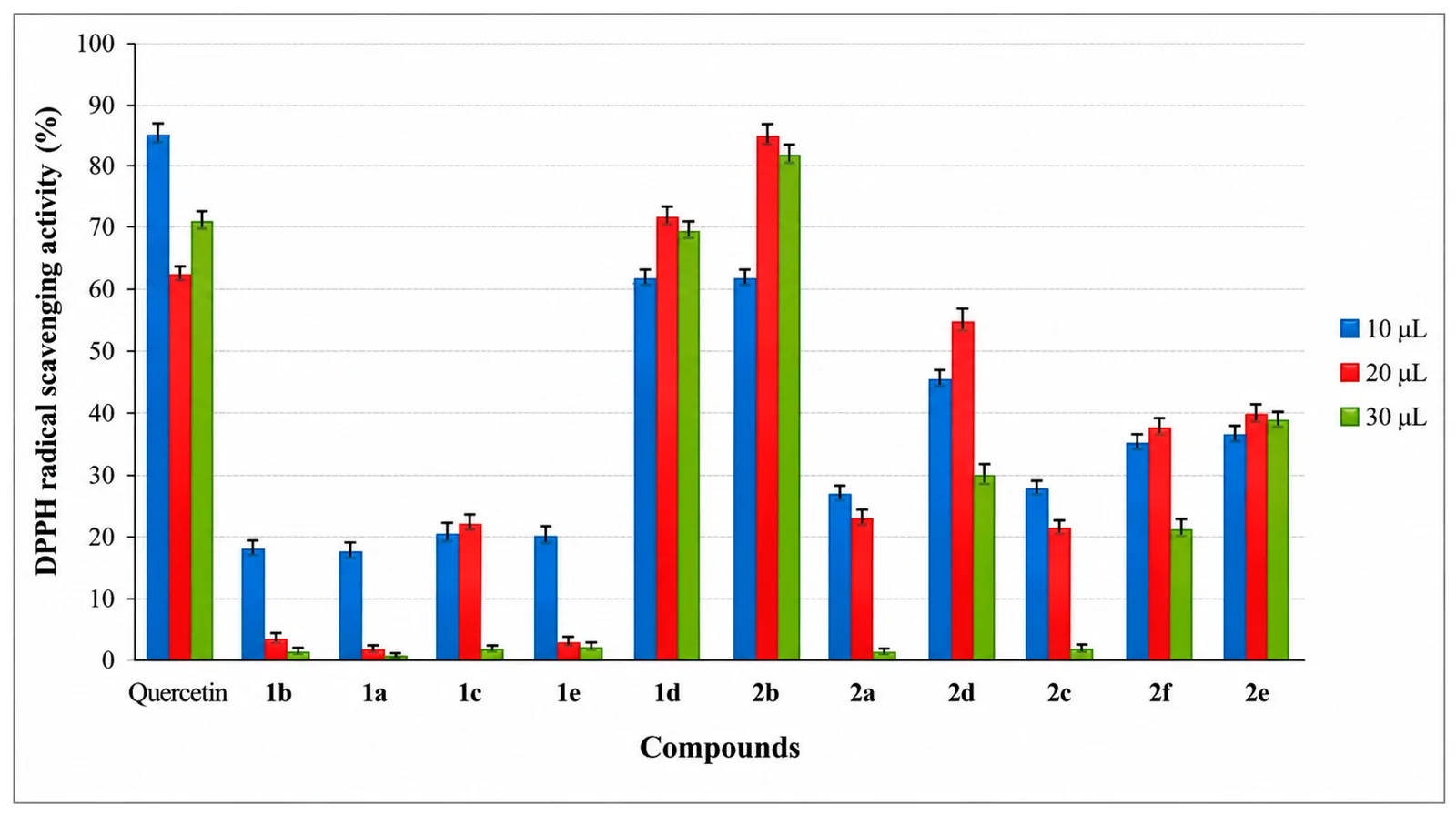
DPPH radical-scavenging activities of the tested benzofuran derivatives (**1a**–**1e** and **2a**–**2f**) and quercetin at treatment volumes of 10, 20, and 30 µL. The corresponding compound-specific final molar concentrations (µM) are provided in the Materials and Methods section. Data are presented as mean ± standard deviation (SD).

DPPH radical-scavenging responses varied across compounds and treatment levels (Figure 2). Quercetin showed comparatively high activity at all three levels. Among the tested derivatives, **1d** and **2b** showed comparatively high mean responses, particularly at 20 and 30 µL. Responses did not uniformly increase: **2d** showed a higher mean activity at 20 µL followed by a decrease at 30 µL, while **2a** and **2c** showed lower mean activities with increasing treatment volume. Compound **2e** showed similar mean responses across the three levels, and **2f** showed a lower response at 30 µL than at 10 and 20 µL.

Compound-specific final molar concentrations are provided in the Materials and Methods section. Equal treatment volumes represented different molar exposures; therefore, the observed mean responses were not used to rank the derivatives by molar radical-scavenging potency. The raw DPPH absorbance values, group means, and normalized radical-scavenging activities underlying Figure 1 are provided in Supplementary Table S1.

### 2.3. Effects of the Tested Compounds on In Vitro LPO

**Figure 3:**
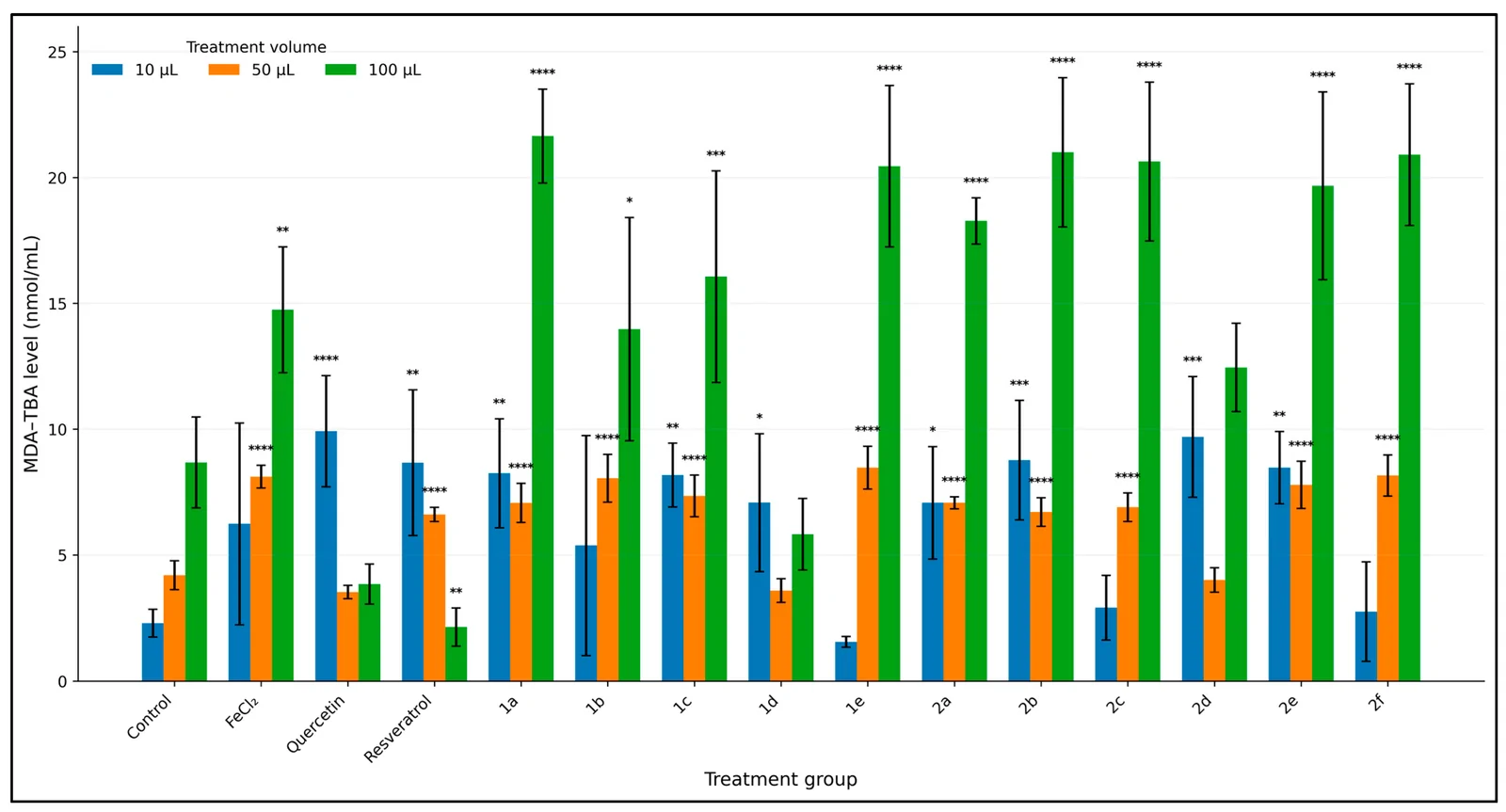
Effects of the tested benzofuran derivatives, quercetin, and resveratrol on MDA–TBA levels in the in vitro LPO assay at treatment volumes of 10, 50, and 100 µL. Data are presented as mean ± SD of five independent measurements (*n* = 5). Statistical comparisons were performed separately at each treatment level using one-way analysis of variance (ANOVA) followed by Dunnett’s multiple-comparisons test against the corresponding control group. *p* < 0.05 (*), *p* < 0.01 (**), *p* < 0.001 (***), and *p* < 0.0001 (****).

The in vitro TBARS assay showed compound- and treatment-dependent differences in MDA–TBA levels at the three treatment volumes (Figure 3). The corresponding final molar concentrations are provided in the Materials and Methods section.

Control MDA–TBA levels were 2.297 ± 0.552, 4.195 ± 0.573, and 8.675 ± 1.806 nmol/mL at the 10, 50, and 100 µL treatment levels, respectively. At 10 µL, levels were significantly higher than the corresponding control for quercetin, resveratrol, **1a**, **1c**, **1d**, **2a**, **2b**, **2d**, and **2e**. At 50 µL, significant increases were observed for FeCl_2_, resveratrol, **1a**, **1b**, **1c**, **1e**, **2a**, **2b**, **2c**, **2e**, and **2f**, whereas quercetin, **1d**, and **2d** did not differ significantly from the control.

At 100 µL, MDA–TBA levels were significantly increased for FeCl_2_ and all tested derivatives except **1d** and **2d**. Resveratrol significantly decreased MDA–TBA levels (*p* < 0.01), whereas quercetin, **1d**, and **2d** did not differ significantly from the corresponding control. Thus, the responses varied across treatment levels rather than showing a uniform increase or decrease.

The raw absorbance values, group means, and normalized MDA values underlying Figure 2 are provided in Supplementary Table S2.

### 2.4. Effects of the Tested Compounds on Intracellular GSH, MDA, and Total Protein Levels

Intracellular GSH, MDA, and total protein results are presented in Table 1, Table 2, Table 3 and Table 4.

In Experimental Set 1, **2e** increased both GSH and MDA (*p* < 0.05), without significantly altering total protein. Compound **1a** increased total protein (*p* < 0.05), while GSH and MDA did not differ significantly from the control.

In Experimental Set 2, **1e**, **1d**, and **2f** decreased GSH (*p* < 0.001), while MDA remained statistically unchanged. The GSH level following **2f** treatment was 0.01 ± 0.01 µmol/g wet pellet compared with 0.73 ± 0.03 µmol/g wet pellet in the control. Total protein decreased with **1e** (*p* < 0.05) and **1d** (*p* < 0.01), but increased with **2f** (*p* < 0.01).

In Experimental Set 3, **1c** increased GSH and total protein (*p* < 0.01), while **2d** and **2c** did not significantly alter GSH. None of the treatments significantly altered MDA. Total protein increased with **2d** (*p* < 0.001) and decreased with **2c** (*p* < 0.05).

In Experimental Set 4, **1b** and **2b** decreased GSH (*p* < 0.01) and increased MDA (*p* < 0.05 and *p* < 0.01, respectively). Total protein increased with **2b** (*p* < 0.05), while the change with **1b** was not significant.

### 2.5. Effects of the Tested Compounds on the Fatty Acid Profile of S. cerevisiae

Fatty acid profiles are presented in Table 5, Table 6, Table 7 and Table 8. The significant treatment-associated changes within each experimental set are summarized below.

In Experimental Set 1, both **1a** and **2e** increased palmitoleic acid (16:1). Their oleic acid (18:1n-9) responses differed in direction: **1a** decreased this fatty acid, whereas **2e** increased it. Compound **1a** also decreased stearic acid (18:0), while **2e** decreased arachidic acid (20:0). The corrected palmitic acid (16:0) value for **2e** was 99.89 ± 7.45 µg/g wet pellet; its statistical significance was not assessed because the required replicate-level data were unavailable.

In Experimental Set 2, all three treatments increased palmitic acid (16:0), stearic acid (18:0), and trans-oleic acid (18:1n-9t), while decreasing palmitoleic acid (16:1). Cis-oleic acid (18:1n-9c) increased only with **1d**. Linoleic acid (18:2n-6c) decreased with **1e** and increased with **2f**, but was not significantly altered by **1d**. Lauric acid (12:0) decreased with **1d** and **2f**.

In Experimental Set 3, **2d** and **2c** increased palmitic acid (16:0), palmitoleic acid (16:1), and trans-oleic acid (18:1n-9t). Compound **1c** also increased palmitoleic acid but decreased palmitic acid, stearic acid (18:0), and trans- and cis-oleic acid fractions. Lauric acid (12:0) decreased with **2d** and **2c** and was not detected following **1c** treatment. Neither cis- nor trans-linoleic acid differed significantly from the control following any treatment.

In Experimental Set 4, both **1b** and **2b** increased palmitic acid (16:0), palmitoleic acid (16:1), stearic acid (18:0), arachidic acid (20:0), erucic acid (22:1), and docosadienoic acid (22:2). Cis-oleic acid (18:1n-9c) increased with **1b** but decreased with **2b**. Lauric acid (12:0) decreased only with **2b**.

### 2.6. Effects of the Tested Compounds on Lipophilic Vitamin and Sterol Profiles in S. cerevisiae

Lipophilic vitamin and sterol profiles are presented in Table 9, Table 10, Table 11 and Table 12.

In Experimental Set 1, both **2e** and **1a** decreased α-tocopherol and stigmasterol and increased ergosterol. Compound **2e** additionally increased vitamin K_1_ and decreased β-sitosterol. Neither treatment significantly altered δ-tocopherol, vitamin D_2_, or vitamin K_2_.

In Experimental Set 2, α-tocopherol increased with **1d** and **2f**, while vitamin K_2_ decreased with **1e** and **1d**. All three treatments increased stigmasterol, without significantly altering ergosterol or β-sitosterol. No significant changes were detected in δ-tocopherol, vitamin D_2_, or vitamin K_1_.

In Experimental Set 3, all three treatments increased α-tocopherol. Stigmasterol increased with **2d** and **2c**, while **2c** additionally increased ergosterol and decreased β-sitosterol. δ-Tocopherol decreased with **2d** and **1c**. None of the treatments significantly altered vitamin D_2_, vitamin K_1_, or vitamin K_2_.

In Experimental Set 4, **1b** increased α-tocopherol, δ-tocopherol, vitamin D_2_, ergosterol, and stigmasterol. Compound **2b** decreased stigmasterol and increased β-sitosterol, without significantly altering the other statistically evaluated parameters. The corrected vitamin K_1_ values were 0.25 ± 0.12, 0.15 ± 0.11, and 0.10 ± 0.06 µg/g wet pellet for the control, **1b**, and **2b** groups, respectively; statistical significance was not assessed for these values because the required replicate-level data were unavailable.

## 3. Discussion

The principal finding of this study is that chemical radical-scavenging responses did not consistently correspond to the cellular redox-related responses of the tested benzofuran derivatives. Changes in GSH and MDA were also not consistently parallel, while fatty acid, lipophilic vitamin, and sterol measurements revealed additional treatment-associated differences. Together, these endpoints provide a broader biochemical characterization than the DPPH assay alone. Cellular findings are interpreted relative to the control of each experimental set, rather than as a ranking of compounds across sets.

The DPPH results showed variable and sometimes non-linear radical-scavenging responses, with **1d** and **2b** displaying comparatively high mean activities under the tested conditions. Similar compound-dependent behavior has been reported for benzofuran-2-one, chalcone, and pyrazoline derivatives [34,35,36]. Molecular features affecting electron or hydrogen donation may contribute to these differences [37,38]. The hydroxyl groups of quercetin provide a relevant example of how the number, position, and chemical environment of potential hydrogen-donating groups can influence radical-scavenging behavior [39,40]. Solvent conditions, electronic properties, and accessibility to the DPPH radical may also affect assay responses [41,42], although these factors were not independently evaluated here.

The non-linear profiles indicate that increasing treatment volume did not consistently increase the measured response. DPPH measurements are sensitive to reaction kinetics and compound-specific reaction behavior [43]. In addition, equal treatment volumes corresponded to different molar concentrations, limiting comparisons of relative potency. The present findings therefore describe chemical assay responses under the tested conditions and do not establish which structural features caused them or whether they translate into cellular antioxidant effects.

The variable DPPH responses were accompanied by treatment-dependent responses in the separate in vitro lipid-containing system, where the derivatives did not consistently suppress MDA–TBA formation. At the highest treatment level, most derivatives increased MDA–TBA levels, whereas **1d** and **2d** did not differ significantly from the control and resveratrol decreased them. Previous yeast studies have also reported variation in LPO-related responses with treatment and experimental conditions [44,45,46], although their cellular findings are not directly equivalent to those obtained in the present cell-free system.

Interpretation also depends on assay specificity. TBA-based methods detect reactive substances other than MDA [47,48,49]; consequently, the measured changes cannot be attributed exclusively to MDA formation. Possible contributions from solubility, aggregation, or interactions within the reaction mixture remain untested. These results therefore characterize changes in the TBARS response without establishing a defined antioxidant or pro-oxidant mechanism.

Moving from the cell-free assays to the yeast model, the cellular findings further illustrate the need to distinguish chemical radical-scavenging responses from intracellular biochemical changes. Compound **1d** showed comparatively high DPPH activity but decreased cellular GSH without significantly altering MDA. Similarly, **2b** showed comparatively high DPPH activity while decreasing GSH and increasing MDA relative to its cellular control. These observations do not demonstrate a quantitative relationship between assays, but they show that a comparatively high DPPH response was not sufficient to identify a consistent cellular response.

The combinations of GSH and MDA responses were also informative. Compound **2e** increased both parameters, whereas **2f** markedly decreased GSH without significantly altering MDA. In contrast, **1b** and **2b** decreased GSH while increasing MDA. The marked GSH decrease with **2f** is therefore a notable biochemical observation, but its occurrence without a significant MDA change prevents interpreting it as evidence of increased lipid peroxidation on its own.

GSH and MDA describe different aspects of cellular biochemical status. GSH abundance can change through synthesis, utilization, and regeneration, while MDA-related measurements can be influenced by lipid substrate availability and cellular metabolic conditions [50,51,52]. Izawa et al. (1995) showed that depletion of intracellular glutathione or inhibition of its synthesis increased the sensitivity of *S. cerevisiae* to H_2_O_2_ and impaired adaptation, whereas increasing intracellular glutathione through precursor supplementation enhanced resistance [53]. This finding establishes a role for glutathione in the response to a defined oxidative challenge. However, the functional role of glutathione under a defined H_2_O_2_ challenge cannot be directly inferred from the GSH concentration measured after exposure to the present derivatives.

The simultaneous increases in GSH and MDA with **2e** are compatible with, but do not establish, an adaptive glutathione response occurring alongside increased formation of lipid peroxidation-related products. Glutathione depletion may arise through several processes and should not, on its own, be equated with increased lipid peroxidation [54,55,56]. The different combinations observed here support describing distinct biochemical response profiles rather than assigning a common antioxidant or pro-oxidant effect to the series [57,58,59].

Total protein measurements added another dimension to the cellular response: **1e**, **1d**, and **2f** all decreased GSH, but their protein responses differed. Total protein decreased with **1e** and **1d** but increased with **2f**. Changes in total protein can be associated with protein synthesis or degradation, metabolic state, growth, and cellular stress [60,61,62]. Because the values are normalized to wet pellet mass, they describe protein content per unit wet biomass and should not be interpreted as direct measurements of culture growth or total biomass production.

Fatty acid profiling added information not captured by GSH and MDA measurements alone. Treatments with **1e**, **1d**, and **2f** decreased palmitoleic acid while increasing palmitic and stearic acids relative to their control. Other treatments produced different patterns: **1a** and **2e** increased palmitoleic acid but showed opposite directional changes in oleic acid. Dilsiz et al. (1997) examined the effects of selenium, vitamin E, and their combination on fatty acid composition and protein measurements in *S. cerevisiae* [63], providing a relevant example of evaluating these biochemical endpoints together. You et al. (2003) demonstrated that cellular oleic acid content was an important determinant of ethanol tolerance in *S. cerevisiae* [64], while Sajbidor et al. (1995) examined the effects of ethanol on yeast lipid content and fatty acid composition [65]. These studies provide context for interpreting treatment-associated fatty acid changes. In the present study, **1b** and **2b** produced opposite directional changes in cis-oleic acid relative to their control, but ethanol tolerance and membrane function were not assessed; the physiological consequences of these changes therefore remain unresolved.

Fatty acid synthesis, elongation, and lipid turnover contribute to the regulation of yeast lipid composition [66,67]. Stukey et al. (1989) characterized OLE1, a gene affecting fatty acid desaturation in *S. cerevisiae*, providing a biological basis for considering desaturation when interpreting saturated and monounsaturated fatty acid profiles [68]. Changes in measured fatty acid abundance may also involve turnover or incorporation into different lipid classes. Because the present measurements were expressed as µg/g wet pellet and did not resolve lipid classes or membrane incorporation, they do not establish altered OLE1 expression, desaturase activity, or membrane fluidity.

The increases in the reported C22:1 and C22:2 fractions following **1b** and **2b** treatment warrant further investigation. Pedroso et al. (2009) examined changes in plasma membrane lipid profiles and microdomains following H_2_O_2_ exposure in *S. cerevisiae* [69], providing context for studying lipid composition under oxidative challenge. More specifically, Rössler et al. (2003) investigated genes involved in very-long-chain fatty acid synthesis in *S. cerevisiae* [70], providing a basis for considering elongation-related processes. Komori et al. (2023) characterized lipidome variation among industrial *S. cerevisiae* strains using untargeted lipidomics [71], illustrating the importance of broader lipid characterization. However, none of these studies establishes the origin of the C22 changes observed here, and increased abundance alone does not demonstrate activation of an elongation pathway. Targeted lipidomic and molecular analyses would be needed to clarify the identity, distribution, and metabolic origin of these fractions [72,73,74].

Beyond fatty acids, the lipophilic vitamin and sterol measurements revealed additional differences in the biochemical response profiles. Compounds **1a** and **2e** increased ergosterol while decreasing α-tocopherol and stigmasterol. In contrast, several other treatments increased α-tocopherol or stigmasterol without significantly altering ergosterol. A particularly informative example was provided by **1b** and **2b**: both decreased GSH and increased MDA, but **1b** increased tocopherols, ergosterol, and stigmasterol, whereas **2b** decreased stigmasterol and increased β-sitosterol. Thus, similar directional changes in GSH and MDA did not imply an identical lipophilic profile.

Previous studies have examined sterol-associated processes in yeast, including the physiological implications of sterol biosynthesis [75] and the effects of fluconazole on membrane rigidity, heterogeneity, and water penetration [76]. These studies support considering sterol measurements alongside functional membrane endpoints. In the present study, compounds **1b** and **2b** showed different directional changes in selected sterol measurements relative to their shared control, but membrane function was not assessed. Related chalcone and pyrazoline frameworks have been investigated for biological activity [77,78,79], providing context for further study of these response profiles. However, **1b** and **2b** were not directly compared statistically, and their observed responses cannot be attributed specifically to the difference between the chalcone and pyrazoline frameworks.

Abe and Hiraki (2009) investigated the relationship between ergosterol, membrane rigidity, and cycloheximide resistance in *S. cerevisiae* [80]. Liu et al. (2013) reported that D-limonene exposure decreased intracellular ergosterol while increasing membrane fluidity, permeability, and the saturated fatty acid ratio [81]. These studies illustrate the value of assessing sterol abundance together with functional membrane endpoints. In the present study, ergosterol increased following several treatments, but membrane properties were not measured; these increases therefore cannot be interpreted as evidence of improved membrane integrity. The regulation of ergosterol biosynthesis provides additional context [82], although pathway activity was not directly assessed here. The measured tocopherol, stigmasterol, and β-sitosterol values likewise describe their abundance in the analyzed fractions; they do not establish endogenous synthesis by yeast.

Previous studies have evaluated chalcone and pyrazoline derivatives through computational analyses, anticancer assays, and enzyme-inhibition measurements [83,84,85]. These investigations provide context for their biological activity but do not explain the biochemical responses observed in the present yeast model. In addition, unequal molar exposures and the evaluation of compounds in separate experimental sets limit comparative interpretation of the present findings. The data therefore characterize individual treatment-associated response profiles without establishing a structure–activity relationship across the series.

The interpretation of these response profiles also requires consideration of possible effects on cell viability. Following the synthesis studies of Coşkun et al. (2008,2011) [32,33], Avci et al. (2023) [86] examined selected benzofuran-derived chalcones in human cancer and non-malignant cell lines, reporting concentration-dependent anti-growth effects and conducting complementary cell-death analyses. These findings support further cellular investigation but cannot substitute for viability measurements in the present yeast model, where the exposure conditions and biological endpoints differed.

The contribution of this study is the joint characterization of chemical assay responses and cellular redox-related and lipid-associated endpoints for the tested derivatives. Chemical radical-scavenging capacity was insufficient on its own to characterize the cellular response, and GSH changes required consideration alongside MDA and the broader biochemical profile. Forman et al. (2014) distinguished radical-scavenging reactions measured in vitro from biological antioxidant defense, emphasizing that chemical reactivity alone does not establish an effective antioxidant action in vivo [87]. The broader physiological roles of ROS and antioxidants provide further context for this distinction [88]. The present divergence between DPPH responses and cellular GSH–MDA patterns is consistent with treating chemical and cellular endpoints separately, but it does not identify the processes responsible for that divergence. The findings identify specific response combinations for further investigation rather than demonstrating a common antioxidant mechanism.

### 3.1. Study Limitations and Future Directions

The cellular experiments were conducted in four independent sets with separate controls, and the treatments were matched by mass rather than molar concentration. Consequently, the results support treatment-versus-control interpretation within each set but do not establish relative potency or structure–activity relationships across the series. Future comparisons should use a unified design with matched molar concentrations, exposure periods, and culture conditions.

Cell viability, growth kinetics, membrane integrity, intracellular ROS, GSSG/GSH status, and antioxidant enzyme activities were not measured concurrently. The observed biochemical changes therefore cannot distinguish adaptive responses from altered growth, nonspecific stress, or cytotoxicity. Previous mammalian findings [86] do not resolve this limitation. Concurrent viability and redox measurements are needed to clarify the cellular significance of the GSH–MDA response combinations.

The DPPH assay measures a cell-free chemical response, while TBARS assays have limited specificity for MDA. Replicate-level DPPH data were unavailable for retrospective inferential analysis; the DPPH findings are therefore descriptive. Statistical significance was also not assessed for the corrected 16:0 value for **2e** or the corrected vitamin K_1_ values in Experimental Set 4 because the required replicate-level data were unavailable.

Fatty acid, vitamin, and sterol measurements did not resolve metabolic flux, lipid classes, intracellular distribution, or pathway-specific activity. They cannot establish lipid biosynthetic or membrane-remodeling mechanisms. Further work should combine chromatographic confirmation and targeted lipid analyses with cellular and molecular measurements. Validation in mammalian models would also be required before broader biological relevance could be established.

### 3.2. Conclusions

The tested benzofuran–chalcone, benzofuran–pyrazoline, and bis-pyrazoline derivatives produced distinct chemical and cellular biochemical response profiles. Comparatively high DPPH responses were not consistently accompanied by a common cellular GSH–MDA pattern, and GSH changes were not uniformly associated with MDA changes. Fatty acid, lipophilic vitamin, and sterol measurements provided complementary information, including differences between treatments with similar directional redox-related responses. This study therefore supports evaluating these endpoints together when characterizing the biochemical effects of the tested derivatives. The findings remain descriptive with respect to mechanism and cross-compound potency, pending unified molar exposure designs and concurrent viability and molecular measurements.

## 4. Materials and Methods

### 4.1. Chemicals and Reagents

All chemicals and reagents used in the experiments were of analytical or chromatographic grade, as appropriate. 2,2-Diphenyl-1-picrylhydrazyl (DPPH), 5,5′-dithiobis(2-nitrobenzoic acid) (DTNB), GSH, quercetin, resveratrol, linoleic acid, TBA, trichloroacetic acid (TCA), butylated hydroxytoluene (BHT), Folin–Ciocalteu reagent, bovine serum albumin (BSA, Albumin Fraction V), and the analytical standards used for fatty acid, vitamin, and sterol analyses were obtained from Sigma-Aldrich (St. Louis, MO, USA).

Methanol, ethanol, acetonitrile, n-hexane, 2-propanol, n-butanol, dimethyl sulfoxide (DMSO), glacial acetic acid, and other solvents used for sample preparation and chromatographic analyses were obtained from Sigma-Aldrich (St. Louis, MO, USA) and were of analytical or high-performance liquid chromatography (HPLC) grade, as appropriate. Distilled water was used for the preparation of aqueous solutions.

### 4.2. Tested Compounds and General Experimental Design

The compounds evaluated in this study were previously synthesized and characterized according to the procedures reported by Coşkun et al. [32,33]. The series comprised benzofuran–chalcone derivatives **1a**–**1e**, benzofuran–pyrazoline derivatives **2a**–**2e**, and the bis-pyrazoline derivative **2f**. Their structures and compound labels are presented in Figure 1.

The experimental approach comprised three components: a cell-free DPPH radical-scavenging assay, an in vitro TBARS assay using a linoleic acid-containing reaction system, and biochemical analyses of treated *S. cerevisiae* cultures. Cellular measurements included GSH, MDA–TBA, total protein, fatty acids, and lipophilic vitamins and sterols. Assay-specific preparation and exposure conditions are described below.

Cellular experiments were conducted in four separate experimental sets, each with a concurrently prepared vehicle control. Set 1 included **2e** and **1a**; Set 2 included **1e**, **1d**, and **2f**; Set 3 included **2d**, **2c**, and **1c**; and Set 4 included **1b** and **2b**. Each set comprised three independent biological replicates per group. Statistical comparisons were restricted to treatments versus their corresponding control within each set, as described in Section 4.16.

### 4.3. Preparation of Saccharomyces cerevisiae Cultures

Commercial fresh baker’s yeast (Yuva, Türkiye) was used as the source of *S. cerevisiae*. Stock cultures were established by repeated aseptic streaking on nutrient agar plates and incubation at 30 °C for 24 h. Cultures were stored at 4 °C and refreshed at 20-day intervals.

Yeast extract–peptone–dextrose (YEPD) medium contained 1% (*w*/*v*) yeast extract, 2% (*w*/*v*) bactopeptone, and 2% (*w*/*v*) glucose. The medium was sterilized by autoclaving at 121 °C for 15 min [89,90].

For inoculum preparation, a loopful of cells from the stock culture was transferred into sterile liquid YEPD medium and incubated at 30 °C for 24 h. The resulting culture was used to inoculate the treatment and vehicle-control cultures.

### 4.4. Treatment of Yeast Cultures with the Tested Compounds

Individual compound stock solutions were prepared in DMSO at 2 mg/mL. Each treatment culture received 1 mL of the 24 h inoculum and 50 µL of the corresponding stock solution, equivalent to 100 µg of compound. The final culture volume was adjusted to 10 mL, giving a nominal compound concentration of 10 µg/mL and a DMSO concentration of 0.5% (*v*/*v*).

Vehicle-control cultures received the same inoculum volume and 50 µL of DMSO without compound. Cultures were incubated at 30 °C for 72 h before harvesting. Compound-specific nominal molar concentrations are provided in Table 13.

### 4.5. DPPH Radical-Scavenging Assay

DPPH radical-scavenging activity was measured using a previously described method [91]. DPPH was prepared in methanol at 25 mg/L. Aliquots of 10, 20, or 30 µL of each 2 mg/mL compound stock solution were added to 4 mL of DPPH solution. Vehicle controls contained the same DPPH solution and the corresponding volume of DMSO without compound. Quercetin was evaluated as the reference compound under the same conditions.

The final assay volumes were 4.010, 4.020, and 4.030 mL, respectively. Compound-specific final molar concentrations are provided in Table 13.

Reaction mixtures were incubated in the dark at room temperature for 30 min, and absorbance was measured at 517 nm. DPPH radical-scavenging activity was calculated using Equation (1):

Inhibition (%) = 100·(A_control_ − A_sample_)/A_control_ (1)

where *A*_control_ is the absorbance of the volume-matched vehicle control and *A*_sample_ is the absorbance of the corresponding compound-containing reaction mixture.

### 4.6. In Vitro LPO Assay

In vitro lipid peroxidation-related responses were evaluated in a linoleic acid-containing reaction system using a modified TBARS approach. The TBA-based measurement was informed by the method of Ohkawa et al. (1979) [92], while previous studies provided methodological context for assessing antioxidant activity using TBARS and in vitro lipid peroxidation models [93,94]. Quercetin and resveratrol were included as reference compounds. Individual compound stock solutions were prepared in DMSO at 2 mg/mL.

A diluted hydrogen peroxide (H_2_O_2_) solution was prepared by adding 10 µL of the stock H_2_O_2_ solution and adjusting the final volume to 100 mL with distilled water. Stock solutions of the tested compounds were prepared individually in DMSO at a concentration of 2 mg/mL. Quercetin and resveratrol were used as reference compounds and were prepared and evaluated under the same experimental conditions.

Each treatment mixture contained 5 mL of buffer, 1 mL of FeCl_2_ solution, 20 µL of linoleic acid, 1 mL of diluted H_2_O_2_ solution, and 10, 50, or 100 µL of compound stock solution. These aliquots supplied 20, 100, or 200 µg of compound and produced final reaction volumes of 7.030, 7.070, or 7.120 mL, respectively. Compound-specific final molar concentrations are provided in Table 14. Quercetin and resveratrol were tested at the same stock concentration and aliquot volumes.

The non-oxidative control, labeled “Control” in Figure 3, contained 5 mL of buffer and 20 µL of linoleic acid, without FeCl_2_, H_2_O_2_, or test compound. The omitted FeCl_2_ and H_2_O_2_ solution volumes were replaced with an equal volume of distilled water (2 mL). The oxidative control, labeled “FeCl_2_ + H_2_O_2_” in Figure 3, contained 5 mL of buffer, 1 mL of FeCl_2_ solution, 20 µL of linoleic acid, and 1 mL of diluted H_2_O_2_ solution, without the test compound. Both controls received 10, 50, or 100 µL of DMSO at the corresponding treatment level, resulting in matched final volumes of 7.030, 7.070, and 7.120 mL, respectively.

Reaction mixtures were vortexed and incubated at 37 °C for 24 h. A 4% (*w*/*v*) BHT solution was prepared in DMSO. After incubation, 100 µL of this solution was added to each mixture to limit further oxidation during subsequent sample processing.

The TBA reagent was prepared by combining 4 g of NaOH, 10 mL of glacial acetic acid, and 0.67 g of TBA with approximately 60 mL of distilled water. The mixture was heated until the components dissolved, cooled to room temperature, and made up to a final volume of 100 mL with distilled water.

For TBARS determination, 1 mL of each incubated reaction mixture was combined with 1 mL of TBA reagent and 1 mL of distilled water in a capped tube. Samples were vortexed and heated at 95 °C for 45 min. After cooling to room temperature, 1 mL of n-butanol was added. Samples were vortexed and centrifuged at 4500 rpm for 5 min, and the upper organic phase was collected for absorbance measurement at 532 nm.

MDA–TBA levels were calculated using the calibration equation y = 0.0492x − 0.0344, where y is absorbance at 532 nm and x is concentration in nmol/mL. Accordingly, x was calculated as (y + 0.0344)/0.0492. Five independent replicates were analyzed for each condition (*n* = 5). The measured endpoint was designated as MDA–TBA because the TBA reaction can involve substances other than MDA, as demonstrated by LC-MS investigation of TBARS reaction products [95].

### 4.7. Preparation of Yeast Cell Homogenates

After the 72 h exposure period, yeast cultures were centrifuged at 7000 rpm for 10 min at 4 °C. The supernatant was discarded, and the wet pellet mass was recorded. Cell extracts were prepared using a modified procedure based on previously described yeast extraction methods [96,97].

The cell pellets were resuspended in a cold Tris-based disruption buffer containing Tris base, Tris-HCl, and ethylenediaminetetraacetic acid (EDTA) (pH 7.4) at a ratio of 1 g wet cell pellet to 10 mL of buffer. The pellets were initially dispersed using a glass rod and subsequently homogenized under cold conditions.

The homogenates were centrifuged at 7000 rpm for 10 min at 4 °C. The supernatants were used for GSH, total protein, and intracellular TBARS measurements. The residual pellets were retained for lipid extraction and subsequent fatty acid, lipophilic vitamin, and sterol analyses.

### 4.8. Determination of GSH

GSH was determined using a modified DTNB-based method described by Ellman and Sedlak and Lindsay [98,99]. One milliliter of yeast homogenate supernatant was mixed with 1 mL of 10% (*w*/*v*) TCA, vortexed, and centrifuged at 5000 rpm for 10 min to precipitate proteins.

The resulting supernatant was transferred to a clean tube and mixed with 2 mL of 0.3 M Na_2_HPO_4_ solution and 1 mL of DTNB reagent. Absorbance was measured immediately at 412 nm after development of the yellow reaction product.

Quantification was performed using reduced GSH standards prepared from 20, 40, 60, 80, and 100 µL aliquots of the standard solution and processed under the same assay conditions. The reagent blank contained distilled water, Na_2_HPO_4_ solution, and DTNB reagent without GSH. Results were calculated from the calibration curve, normalized to the initial wet cell pellet mass, and expressed as µmol GSH/g wet pellet.

### 4.9. Determination of Total Protein

Total protein was determined using the Lowry method [100]. A freshly prepared working reagent containing sodium hydroxide, sodium carbonate, copper sulfate, and potassium sodium tartrate was used. Folin–Ciocalteu reagent was diluted 1:1 (*v*/*v*) with distilled water immediately before use.

Five microliters of yeast homogenate supernatant was mixed with 4 mL of Lowry working reagent and incubated at room temperature for 10 min. Subsequently, 0.5 mL of diluted Folin–Ciocalteu reagent was added. Samples were vortexed and incubated for a further 30 min before absorbance measurement at 750 nm.

BSA standards prepared from 20, 40, 60, 80, and 100 µL aliquots of the standard solution were processed under the same conditions. A reagent blank containing the working reagent and diluted Folin–Ciocalteu reagent was used. Protein concentrations were calculated from the BSA calibration curve, normalized to the initial wet cell pellet mass, and expressed as mg protein/g wet pellet.

### 4.10. Determination of Intracellular TBARS

Intracellular lipid peroxidation-related responses were assessed using a modified TBARS method [101,102]. Two milliliters of yeast homogenate supernatant was mixed with 1 mL of cell-disruption buffer, 50 µL of BHT solution, 1 mL of TBA reagent, 1 mL of SDS solution, 1 mL of HCl solution, and 1 mL of 10% (*w*/*v*) TCA.

BHT was prepared at 4% (*w*/*v*) in ethanol, and SDS was prepared at 4.05% (*w*/*v*) in distilled water. The HCl solution was prepared by diluting 4.5 mL of concentrated HCl to 100 mL with distilled water. The preparation of the TBA reagent is described in Section 4.6.

Samples were vortexed, tightly capped, and heated at 110 °C for 1 h. After cooling to room temperature, 3 mL of n-butanol was added. Samples were mixed and centrifuged at 5000 rpm for 10 min. Two milliliters of the upper organic phase was collected, and absorbance was measured at 532 nm against n-butanol. Results were normalized to the initial wet cell pellet mass and expressed as nmol MDA equivalents/g wet pellet.

### 4.11. Extraction of Lipids for Fatty Acid, Vitamin, and Sterol Analyses

Lipids were extracted using n-hexane–2-propanol (3:2, *v*/*v*), following a modified procedure based on Hara and Radin [103]. BHT was added to the extraction solvent at 0.03 g/L.

Ten milliliters of extraction solvent was added to the residual pellet obtained after homogenate centrifugation. Samples were homogenized and centrifuged at 6000 rpm for 15 min. The upper organic phase was collected and divided into portions for fatty acid analysis and lipophilic vitamin and sterol analysis.

### 4.12. Preparation of Samples for Vitamin and Sterol Analysis

A 5 mL aliquot of the lipid extract was mixed with 5 mL of 5% (*w*/*v*) KOH solution, vortexed, and incubated at 85 °C for 15 min. After cooling to room temperature, 5 mL of distilled water was added, and the unsaponifiable fraction was extracted twice with 5 mL of n-hexane [104].

The collected organic extracts were evaporated under nitrogen. The dried residue was reconstituted in 1 mL of acetonitrile–methanol (50:50, *v*/*v*), vortexed, and transferred to an HPLC vial.

### 4.13. Preparation of Fatty Acid Methyl Esters

FAMEs were prepared from the remaining lipid extract by acid-catalyzed transmethylation [105]. Five milliliters of 2% (*v*/*v*) sulfuric acid in methanol was added to each sample. Tubes were tightly capped, vortexed, and incubated at 50 °C for 15 h.

After cooling to room temperature, 5 mL of 5% (*w*/*v*) NaCl solution was added, and FAMEs were extracted with 5 mL of n-hexane. The upper phase was transferred to a clean tube and mixed with 5 mL of 2% (*w*/*v*) KHCO_3_ solution. Samples were maintained at room temperature for 4 h, after which the organic phase was collected and evaporated under nitrogen at 45 °C. The residue was reconstituted in 1 mL of n-hexane and transferred to a GC vial [105].

### 4.14. Gas Chromatographic Analysis of Fatty-Acid Methyl Esters

FAMEs were analyzed using a Shimadzu GC-17 gas chromatograph (Shimadzu, Kyoto, Japan), following a modified procedure [106]. Separation was performed on a PERMABOND capillary column (25 m × 0.25 mm internal diameter; 0.25 µm film thickness; MACHEREY-NAGEL GmbH & Co. KG, Düren, Germany).

The oven temperature was increased from 120 to 200 °C at 5 °C/min and then to 220 °C at 4 °C/min. The final temperature was maintained for 8 min. Injector and detector temperatures were 240 and 280 °C, respectively, and nitrogen was used as the carrier gas.

Fatty acids were identified by comparing sample retention times with those of authentic FAME standards analyzed under the same conditions. Quantification was performed using calibration data from the corresponding standards. Results were normalized to the initial wet cell pellet mass and expressed as µg/g wet pellet.

### 4.15. HPLC Analysis of Lipophilic Vitamins and Sterols

Lipophilic vitamins and sterols were analyzed by reversed-phase HPLC using a modified procedure based on previously reported methods [107,108]. The HPLC system comprised an LC-10ADVP pump, SPD-10AVP UV–Vis detector, CTO-10ASVP column oven, SIL-10ADVP autosampler, and DGU-14A degasser (Shimadzu, Kyoto, Japan).

Separation was performed on a Supelcosil LC-18 column (150 × 4.6 mm, 5 µm; Supelco, Bellefonte, PA, USA). The mobile phase was acetonitrile–methanol (60:40, *v*/*v*), delivered at 1.0 mL/min. Samples were prepared as described in Section 4.12. Tocopherols and sterols were monitored at 202 nm and vitamins D and K at 265 nm.

Analytes were identified by retention-time comparison with authentic standards analyzed under the same conditions and quantified using the corresponding standard calibrations. Results were normalized to the initial wet cell pellet mass and expressed as µg/g wet pellet.

### 4.16. Statistical Analysis

Cellular data were analyzed using IBM SPSS Statistics for Windows, version 22.0 (IBM Corp., Armonk, NY, USA). Each group comprised three independent biological replicates (*n* = 3). The four experimental sets were analyzed separately using one-way ANOVA followed by Dunnett’s multiple-comparisons test against the corresponding vehicle control. No cross-set statistical comparisons were performed.

The in vitro LPO assay comprised five independent replicates per condition (*n* = 5). Data were analyzed separately at each treatment level using one-way ANOVA followed by Dunnett’s test against the corresponding control. The analysis used for Figure 3 was performed using Python (version 2.6; Python Software Foundation, Wilmington, DE, USA) with SciPy (version 0.10; SciPy Community; developed by an international open-source community).

DPPH results are presented descriptively as mean ± SD. Replicate-level DPPH data were unavailable for retrospective inferential analysis; therefore, no new statistical significance designations were assigned. Statistical significance was also not assessed for the corrected 16:0 value for **2e** or the corrected vitamin K_1_ values in Experimental Set 4 because the required replicate-level data were unavailable.

Results are presented as mean ± SD. Statistical significance was defined as *p* < 0.05, with symbols indicating *p* < 0.05 (*), *p* < 0.01 (**), *p* < 0.001 (***), and *p* < 0.0001 (****). “ns” denotes a non-significant difference from the corresponding control.

## Figures and Tables

**Table 1:** Effects of compounds **2e** and **1a** on intracellular GSH, MDA, and total protein levels in *S. cerevisiae*—Experimental Set 1.

Parameter	Control	2e	1a
GSH (µmol/g wet pellet)	1.42 ± 0.25	2.30 ± 0.32 *	1.71 ± 0.19 ns
MDA (nmol/g wet pellet)	4.09 ± 0.37	5.22 ± 0.35 *	3.89 ± 0.34 ns
Total protein (mg/g wet pellet)	5.92 ± 1.20	8.38 ± 1.36 ns	8.79 ± 1.30 *

Data are presented as mean ± SD from three independent biological replicates (*n* = 3). ns, not significant; *p* < 0.05 (*) versus the corresponding concurrently prepared control.

**Table 2:** Effects of compounds **1e**, **1d**, and **2f** on intracellular GSH, MDA, and total protein levels in *S. cerevisiae*—Experimental Set 2.

Parameter	Control	1e	1d	2f
GSH (µmol/g wet pellet)	0.73 ± 0.03	0.12 ± 0.05 ***	0.08 ± 0.05 ***	0.01 ± 0.01 ***
MDA (nmol/g wet pellet)	2.98 ± 0.30	3.20 ± 0.30 ns	3.27 ± 0.30 ns	2.97 ± 0.17 ns
Total protein (mg/g wet pellet)	10.10 ± 1.04	7.65 ± 1.01 *	6.80 ± 0.78 **	13.76 ± 0.82 **

Data are presented as mean ± SD from three independent biological replicates (*n* = 3). ns, not significant; *p* < 0.05 (*), *p* < 0.01 (**), and *p* < 0.001 (***) versus the corresponding concurrently prepared control.

**Table 3:** Effects of compounds **2d**, **2c**, and **1c** on intracellular GSH, MDA, and total protein levels in *S. cerevisiae*—Experimental Set 3.

Parameter	Control	2d	2c	1c
GSH (µmol/g wet pellet)	0.36 ± 0.09	0.27 ± 0.03 ns	0.37 ± 0.04 ns	0.76 ± 0.04 **
MDA (nmol/g wet pellet)	3.70 ± 0.93	5.64 ± 1.00 ns	3.71 ± 0.75 ns	3.62 ± 0.42 ns
Total protein (mg/g wet pellet)	8.59 ± 0.46	14.05 ± 0.53 ***	6.58 ± 0.03 *	10.67 ± 0.51 **

Data are presented as mean ± SD from three independent biological replicates (*n* = 3). ns, not significant; *p* < 0.05 (*), *p* < 0.01 (**), and *p* < 0.001 (***) versus the corresponding concurrently prepared control.

**Table 4:** Effects of compounds **1b** and **2b** on intracellular GSH, MDA, and total protein levels in *S. cerevisiae*—Experimental Set 4.

Parameter	Control	1b	2b
GSH (µmol/g wet pellet)	2.34 ± 0.35	0.90 ± 0.31 **	0.63 ± 0.10 **
MDA (nmol/g wet pellet)	3.68 ± 0.09	6.92 ± 1.03 *	4.32 ± 0.13 **
Total protein (mg/g wet pellet)	5.17 ± 0.82	7.83 ± 1.39 ns	9.48 ± 1.47 *

Data are presented as mean ± SD from three independent biological replicates (*n* = 3). ns, not significant; *p* < 0.05 (*) and *p* < 0.01 (**) versus the corresponding concurrently prepared control.

**Table 5:** Effects of compounds **1a** and **2e** on the fatty acid profile of *S. cerevisiae*—Experimental Set 1.

Fatty Acid (µg/g Wet Pellet)	Control	1a	2e
16:0	80.39 ± 10.82	85.49 ± 10.75 ns	99.89 ± 7.45 ^†^
16:1	95.53 ± 9.43	121.72 ± 9.32 *	156.98 ± 9.92 **
18:0	20.73 ± 0.56	17.18 ± 0.57 **	20.52 ± 0.21 ns
18:1n-9	45.83 ± 0.04	45.41 ± 0.05 ***	61.50 ± 0.06 ****
20:0	10.42 ± 0.15	10.41 ± 0.37 ns	5.15 ± 0.17 ****

Data are presented as mean ± SD from three independent biological replicates (*n* = 3). ns, not significant; *p* < 0.05 (*), *p* < 0.01 (**), *p* < 0.001 (***), and *p* < 0.0001 (****) versus the corresponding concurrently prepared control. ^†^ Statistical significance was not assigned to the corrected 16:0 value for compound **2e** because the replicate-level data required for reliable retrospective inferential analysis were unavailable.

**Table 6:** Effects of compounds **1e**, **1d**, and **2f** on the fatty acid profile of *S. cerevisiae*—Experimental Set 2.

Fatty Acid (µg/g Wet Pellet)	Control	1e	1d	2f
12:0	34.73 ± 1.83	35.79 ± 0.97 ns	14.50 ± 2.41 ***	27.96 ± 1.69 **
16:0	191.65 ± 11.57	230.82 ± 11.50 *	273.29 ± 11.49 ***	233.01 ± 11.58 *
16:1	223.98 ± 10.54	96.50 ± 10.23 ***	122.45 ± 10.66 ***	128.78 ± 10.42 ***
18:0	62.14 ± 1.93	76.06 ± 1.33 ***	87.95 ± 1.74 ***	66.63 ± 1.16 *
18:1n-9t	86.56 ± 0.68	158.22 ± 0.78 ****	160.47 ± 0.59 ****	169.82 ± 0.81 ****
18:1n-9c	35.55 ± 10.70	35.35 ± 10.29 ns	63.40 ± 10.72 *	32.32 ± 10.67 ns
18:2n-6c	10.74 ± 0.02	10.28 ± 0.07 **	12.79 ± 0.89 ns	13.02 ± 0.14 **

Data are presented as mean ± SD from three independent biological replicates (*n* = 3). ns, not significant; *p* < 0.05 (*), *p* < 0.01 (**), *p* < 0.001 (***), and *p* < 0.0001 (****) versus the corresponding concurrently prepared control.

**Table 7:** Effects of compounds **2d**, **2c**, and **1c** on the fatty acid profile of *S. cerevisiae*—Experimental Set 3.

Fatty Acid (µg/g Wet Pellet)	Control	2d	2c	1c
12:0	49.49 ± 2.50	28.16 ± 2.09 ***	25.85 ± 2.01 ***	ND
14:0	95.39 ± 2.48	104.26 ± 2.84 *	106.51 ± 2.05 **	44.03 ± 2.42 ***
15:0	71.15 ± 2.22	63.14 ± 1.04 *	69.99 ± 1.81 ns	31.87 ± 1.09 ***
16:0	458.04 ± 4.40	543.83 ± 4.53 ***	554.20 ± 4.55 ***	272.08 ± 4.43 ****
16:1	160.49 ± 4.41	280.23 ± 2.73 ***	286.99 ± 2.78 ***	215.52 ± 2.91 ***
17:1	138.34 ± 21.47	121.40 ± 21.32 ns	129.88 ± 22.04 ns	60.98 ± 21.06 *
18:0	80.44 ± 12.77	86.92 ± 12.92 ns	80.91 ± 12.81 ns	43.24 ± 12.62 *
18:1n-9t	172.71 ± 11.65	264.24 ± 12.58 ***	259.32 ± 12.92 **	121.42 ± 11.65 **
18:1n-9c	72.49 ± 2.31	48.52 ± 2.60 ***	75.39 ± 2.19 ns	38.35 ± 2.45 ***
18:2n-6c	140.14 ± 20.63	148.41 ± 21.33 ns	166.30 ± 21.38 ns	97.89 ± 22.03 ns
18:2n-6t	40.32 ± 12.57	44.24 ± 9.77 ns	44.27 ± 9.17 ns	42.42 ± 9.70 ns

Data are presented as mean ± SD from three independent biological replicates (*n* = 3). ns, not significant; *p* < 0.05 (*), *p* < 0.01 (**), *p* < 0.001 (***), and *p* < 0.0001 (****) versus the corresponding concurrently prepared control. ND, not detected.

**Table 8:** Effects of compounds **1b** and **2b** on the fatty acid profile of *S. cerevisiae*—Experimental Set 4.

Fatty Acid (µg/g Wet Pellet)	Control	1b	2b
12:0	20.80 ± 1.33	20.52 ± 1.53 ns	14.19 ± 0.95 **
16:0	122.95 ± 2.29	189.16 ± 2.59 ***	128.48 ± 2.76 *
16:1	40.10 ± 1.44	244.43 ± 1.52 ****	58.96 ± 1.63 ***
18:0	35.80 ± 1.93	53.68 ± 1.96 ****	41.60 ± 1.50 ***
18:1n-9c	80.67 ± 1.52	88.38 ± 1.74 *	58.86 ± 1.60 ****
20:0	6.94 ± 1.11	14.91 ± 0.97 ****	12.76 ± 0.57 ***
22:1	246.21 ± 3.11	347.19 ± 3.42 ***	381.10 ± 4.03 ****
22:2	30.16 ± 1.07	49.08 ± 1.29 ***	50.18 ± 1.46 ***

Data are presented as mean ± SD from three independent biological replicates (*n* = 3). ns, not significant; *p* < 0.05 (*), *p* < 0.01 (**), *p* < 0.001 (***), and *p* < 0.0001 (****) versus the corresponding concurrently prepared control.

**Table 9:** Effects of compounds **2e** and **1a** on lipophilic vitamin and sterol profiles in *S. cerevisiae*—Experimental Set 1.

Parameter (µg/g Wet Pellet)	Control	2e	1a
α-Tocopherol	5.25 ± 0.46	0.70 ± 0.10 **	2.88 ± 1.02 *
δ-Tocopherol	0.90 ± 0.25	0.80 ± 0.29 ns	0.53 ± 0.33 ns
Vitamin D_2_	1.60 ± 0.51	0.68 ± 1.03 ns	0.65 ± 0.78 ns
Vitamin K_1_	0.12 ± 0.08	0.35 ± 0.07 *	0.16 ± 0.06 ns
Vitamin K_2_	0.90 ± 0.56	0.91 ± 0.24 ns	0.78 ± 0.24 ns
Ergosterol	4.09 ± 1.11	13.71 ± 1.40 ***	9.62 ± 1.44 **
Stigmasterol	426.59 ± 11.46	250.62 ± 11.40 ****	376.25 ± 11.60 **
β-Sitosterol	2.20 ± 0.31	0.38 ± 0.34 **	0.64 ± 0.76 ns

Data are presented as mean ± SD from three independent biological replicates (*n* = 3). ns, not significant; *p* < 0.05 (*), *p* < 0.01 (**), *p* < 0.001 (***), and *p* < 0.0001 (****) versus the corresponding concurrently prepared control.

**Table 10:** Effects of compounds **1e**, **1d**, and **2f** on lipophilic vitamin and sterol profiles in *S. cerevisiae*—Experimental Set 2.

Parameter (µg/g Wet Pellet)	Control	1e	1d	2f
α-Tocopherol	2.85 ± 1.51	5.32 ± 0.82 ns	14.61 ± 1.33 ***	7.90 ± 1.36 *
δ-Tocopherol	0.79 ± 0.33	0.89 ± 0.24 ns	0.89 ± 0.19 ns	1.02 ± 0.34 ns
Vitamin D_2_	1.28 ± 0.78	1.81 ± 0.39 ns	1.66 ± 0.60 ns	0.25 ± 0.34 ns
Vitamin K_1_	0.16 ± 0.15	0.46 ± 0.34 ns	0.60 ± 0.48 ns	0.47 ± 0.32 ns
Vitamin K_2_	0.49 ± 0.08	0.28 ± 0.09 *	0.08 ± 0.07 **	0.56 ± 0.07 ns
Ergosterol	3.23 ± 0.88	3.25 ± 1.22 ns	2.72 ± 0.44 ns	5.68 ± 1.40 ns
Stigmasterol	198.40 ± 11.22	240.86 ± 11.41 *	246.16 ± 11.12 **	320.22 ± 11.96 ***
β-Sitosterol	0.52 ± 0.37	0.51 ± 0.22 ns	1.23 ± 0.87 ns	0.83 ± 0.59 ns

Data are presented as mean ± SD from three independent biological replicates (*n* = 3). ns, not significant; *p* < 0.05 (*), *p* < 0.01 (**), and *p* < 0.001 (***) versus the corresponding concurrently prepared control.

**Table 11:** Effects of compounds **2d**, **2c**, and **1c** on lipophilic vitamin and sterol profiles in *S. cerevisiae*—Experimental Set 3.

Parameter (µg/g Wet Pellet)	Control	2d	2c	1c
α-Tocopherol	1.59 ± 0.26	6.53 ± 0.07 ***	3.17 ± 0.18 **	2.46 ± 0.14 *
δ-Tocopherol	0.38 ± 0.08	0.15 ± 0.10 *	0.16 ± 0.20 ns	0.10 ± 0.10 *
Vitamin D_2_	0.02 ± 0.04	0.05 ± 0.12 ns	0.09 ± 0.13 ns	0.01 ± 0.00 ns
Vitamin K_1_	0.22 ± 0.15	0.37 ± 0.11 ns	0.42 ± 0.16 ns	0.36 ± 0.16 ns
Vitamin K_2_	1.15 ± 0.45	1.30 ± 0.26 ns	1.22 ± 0.25 ns	0.56 ± 0.46 ns
Ergosterol	10.25 ± 0.24	12.09 ± 1.17 ns	15.75 ± 1.38 *	8.58 ± 1.35 ns
Stigmasterol	525.61 ± 11.71	917.93 ± 11.11 ****	849.76 ± 11.41 ****	535.51 ± 11.11 ns
β-Sitosterol	0.96 ± 0.21	0.31 ± 0.54 ns	0.31 ± 0.29 *	0.49 ± 0.34 ns

Data are presented as mean ± SD from three independent biological replicates (*n* = 3). ns, not significant; *p* < 0.05 (*), *p* < 0.01 (**), *p* < 0.001 (***), and *p* < 0.0001 (****) versus the corresponding concurrently prepared control.

**Table 12:** Effects of compounds **1b** and **2b** on lipophilic vitamin and sterol profiles in *S. cerevisiae*—Experimental Set 4.

Parameter (µg/g Wet Pellet)	Control	1b	2b
α-Tocopherol	13.96 ± 1.36	27.34 ± 1.13 ***	15.82 ± 1.42 ns
δ-Tocopherol	0.14 ± 0.13	1.37 ± 0.10 ***	0.11 ± 0.15 ns
Vitamin D_2_	0.59 ± 0.01	1.01 ± 0.13 *	0.16 ± 0.33 ns
Vitamin K_1_	0.25 ± 0.12 ^†^	0.15 ± 0.11 ^†^	0.10 ± 0.06 ^†^
Vitamin K_2_	0.22 ± 0.16	0.17 ± 0.13 ns	0.11 ± 0.08 ns
Ergosterol	3.76 ± 0.52	5.93 ± 0.45 **	3.19 ± 0.54 ns
Stigmasterol	274.11 ± 2.09	382.27 ± 2.49 ****	182.55 ± 2.02 ****
β-Sitosterol	0.46 ± 0.10	0.65 ± 0.10 ns	0.99 ± 0.17 *

Data are presented as mean ± SD from three independent biological replicates (*n* = 3). ns, not significant; *p* < 0.05 (*), *p* < 0.01 (**), *p* < 0.001 (***), and *p* < 0.0001 (****) versus the corresponding concurrently prepared control. ^†^ Statistical significance was not assessed for the corrected vitamin K_1_ values because the replicate-level data required for retrospective inferential analysis were unavailable.

**Table 13:** Compound-specific nominal molar concentrations used in the DPPH assay and cellular experiments.

Compound	MW (g/mol)	10 µL (µM)	20 µL (µM)	30 µL (µM)	Cellular Experiments, 10 µg/mL (µM)
**1a**	264	18.90	37.69	56.39	37.88
**1b**	264	18.90	37.69	56.39	37.88
**1c**	264	18.90	37.69	56.39	37.88
**1d**	237	21.04	41.99	62.84	42.19
**1e**	294	16.96	33.84	50.65	34.01
**2a**	268	18.61	37.14	55.56	37.31
**2b**	268	18.61	37.14	55.56	37.31
**2c**	278	17.93	35.80	53.57	35.97
**2d**	278	17.93	35.80	53.57	35.97
**2e**	308	16.20	32.31	48.35	32.47
**2f**	446	11.18	22.31	33.38	22.42
Quercetin	302.24	16.49	32.93	49.25	-

Note: Final test-compound concentrations in the DPPH assay were calculated from the 2 mg/mL stock solutions using the molecular weight of each compound and the corresponding final assay volumes of 4.010, 4.020, and 4.030 mL for the 10, 20, and 30 µL treatment levels, respectively. For the cellular experiments, 50 µL of each 2 mg/mL stock solution was added to a final culture volume of 10 mL, corresponding to a nominal mass-based concentration of 10 µg/mL. Because the compounds had different molecular weights, this mass-based concentration corresponded to different compound-specific final molar concentrations. Quercetin was not evaluated in the cellular experiments.

**Table 14:** Final molar concentrations of the tested benzofuran derivatives, quercetin, and resveratrol used in the in vitro LPO assay.

Compound	MW (g/mol)	10 µL (µM)	50 µL (µM)	100 µL (µM)
**1a**	264	10.78	53.58	106.40
**1b**	264	10.78	53.58	106.40
**1c**	264	10.78	53.58	106.40
**1d**	237	12.00	59.68	118.52
**1e**	294	9.68	48.11	95.54
**2a**	268	10.62	52.78	104.81
**2b**	268	10.62	52.78	104.81
**2c**	278	10.23	50.88	101.04
**2d**	278	10.23	50.88	101.04
**2e**	308	9.24	45.92	91.20
**2f**	446	6.38	31.71	62.98
Quercetin	302.24	9.41	46.80	92.94
Resveratrol	228.24	12.47	61.97	123.07

Note: Final compound concentrations were calculated from the 2 mg/mL stock solutions using compound molecular weights and reaction volumes of 7.030, 7.070, and 7.120 mL for the 10, 50, and 100 µL aliquots, respectively. Equal aliquot volumes corresponded to different molar concentrations.

## Data Availability

The data available to support the findings of this study are available from the corresponding author upon reasonable request. Replicate-level raw data for the DPPH assay are no longer available; therefore, retrospective inferential statistical analysis of the DPPH data could not be performed.

## Supplementary Materials

The following supporting information can be downloaded at https://www.mdpi.com/article/10.3390/molecules31203769/s1: Table S1: Raw DPPH absorbance values, group means, and normalized radical scavenging activities (control = 1.0 for each concentration).; Table S2: Raw TBARS absorbance values, group means, and normalized MDA values (control = 1.0).

## Abbreviations

The following abbreviations are used in this manuscript:

BHT	Butylated hydroxytoluene
BSA	Bovine serum albumin
DMSO	Dimethyl sulfoxide
DPPH	2,2-Diphenyl-1-picrylhydrazyl
DTNB	5,5′-Dithiobis(2-nitrobenzoic acid)
EDTA	Ethylenediaminetetraacetic acid
FAMEs	Fatty acid methyl esters
GC	Gas chromatography
GSH	Reduced glutathione
GSSG	Oxidized glutathione
HCl	Hydrochloric acid
HPLC	High-performance liquid chromatography
LPO	Lipid peroxidation
MDA	Malondialdehyde
ROS	Reactive oxygen species
SDS	Sodium dodecyl sulfate
TBA	Thiobarbituric acid
TBARS	Thiobarbituric acid-reactive substance
TCA	Trichloroacetic acid
UV–Vis	Ultraviolet–visible
YEPD	Yeast extract–peptone–dextrose

## References

[1] Jomova K., Raptova R., Alomar S.Y., Alwasel S.H., Nepovimova E., Kuca K., Valko M. (2023). Reactive oxygen species, toxicity, oxidative stress, and antioxidants: Chronic diseases and aging. Arch. Toxicol..

[2] Deng B., Desai S., Ynalvez L., Aziz M.K., Iliescu C. (2025). Evaluating the shared risk factors between atherosclerotic cardiovascular disease and cancer: How significant is the link?. Expert Rev. Cardiovasc. Ther..

[3] Li L., Guo Z., Zhao Y., Liang C., Zheng W., Tian W., Chen Y., Cheng Y., Zhu F., Xiang X. (2025). The impact of oxidative stress on abnormal lipid metabolism-mediated disease development. Arch. Biochem. Biophys..

[4] Sies H., Jones D.P. (2020). Reactive oxygen species (ROS) as pleiotropic physiological signalling agents. Nat. Rev. Mol. Cell Biol..

[5] Forman H.J., Zhang H. (2021). Targeting oxidative stress in disease: Promise and limitations of antioxidant therapy. Nat. Rev. Drug Discov..

[6] Atkinson J., Epand R.F., Epand R.M. (2008). Tocopherols and tocotrienols in membranes: A critical review. Free Radic. Biol. Med..

[7] Mendonça J.S., Guimarães R.C.A., Zorgetto-Pinheiro V.A., Fernandes C.D.P., Marcelino G., Bogo D., Freitas K.C., Hiane P.A., Melo E.S.P., Vilela M.L.B. (2022). Natural Antioxidant Evaluation: A Review of Detection Methods. Molecules.

[8] Arroyave-Ospina J.C., Wu Z., Geng Y., Moshage H. (2021). Role of Oxidative Stress in the Pathogenesis of Non-Alcoholic Fatty Liver Disease: Implications for Prevention and Therapy. Antioxidants.

[9] Khanam H., Shamsuzzaman (2015). Bioactive benzofuran derivatives: A review. Eur. J. Med. Chem..

[10] Chand K., Rajeshwari, Hiremathad A., Singh M., Santos M.A., Keri R.S. (2017). A review on antioxidant potential of bioactive heterocycle benzofuran: Natural and synthetic derivatives. Pharmacol. Rep..

[11] Rudrapal M., Khan J., Dukhyil A.A.B., Alarousy R.M.I.I., Attah E.I., Sharma T., Khairnar S.J., Bendale A.R. (2021). Chalcone Scaffolds, Bioprecursors of Flavonoids: Chemistry, Bioactivities, and Pharmacokinetics. Molecules.

[12] Jung J.-C., Lee Y., Min D., Jung M., Oh S. (2017). Practical Synthesis of Chalcone Derivatives and Their Biological Activities. Molecules.

[13] Mantzanidou M., Pontiki E., Hadjipavlou-Litina D. (2021). Pyrazoles and pyrazolines as anti-inflammatory agents. Molecules.

[14] Nehra B., Rulhania S., Jaswal S., Kumar B., Singh G., Monga V. (2020). Recent advancements in the development of bioactive pyrazoline derivatives. Eur. J. Med. Chem..

[15] Viegas-Junior C., Danuello A., da Silva Bolzani V., Barreiro E.J., Fraga C.A.M. (2007). Molecular hybridization: A useful tool in the design of new drug prototypes. Curr. Med. Chem..

[16] Miao Y.-H., Hu Y.-H., Yang J., Liu T., Sun J., Wang X.-J. (2019). Natural source, bioactivity and synthesis of benzofuran derivatives. RSC Adv..

[17] Mittal A., Vashistha V.K., Das D.K. (2022). Recent advances in the antioxidant activity and mechanisms of chalcone derivatives: A computational review. Free Radic. Res..

[18] Marella A., Ali M.R., Alam M.T., Saha R., Tanwar O., Akhter M., Shaquiquzzaman M., Alam M.M. (2013). Pyrazolines: A biological review. Mini-Rev. Med. Chem..

[19] Shalaby M.A., Rizk S.A., Fahim A.M. (2023). Synthesis, reactions and application of chalcones: A systematic review. Org. Biomol. Chem..

[20] Al-Qadhi M.A., Yahya T.A.A., Fahim S.H., Ragab F.A.F. (2026). Khellin-derived benzofuran–pyrazoline hybrids as kinase-targeted anticancer agents. ACS Omega.

[21] Hussaini S.M.A., Yedla P., Babu K.S., Shaik T.B., Chityal G.K., Kamal A. (2016). Synthesis and Biological Evaluation of 1,2,3-Triazole Tethered Pyrazoline and Chalcone Derivatives. Chem. Biol. Drug Des..

[22] Ali I., Rafique R., Khan K.M., Chigurupati S., Ji X., Wadood A., Salar U., Almahmoud S.A., Rehman A.U., Felemban S.G. (2023). Synthetic benzofuran-linked chalcones with dual actions: A potential therapeutic approach to manage diabetes mellitus. Future Med. Chem..

[23] Coskun D., Erkisa M., Ulukaya E., Coskun M.F., Ari F. (2017). Novel 1-(7-ethoxy-1-benzofuran-2-yl) substituted chalcone derivatives: Synthesis, characterization and anticancer activity. Eur. J. Med. Chem..

[24] Hassan G.S., Abdel Rahman D.E., Saleh D.O., Abdel Jaleel G.A.R. (2014). Benzofuran-morpholinomethyl-pyrazoline hybrids as a new class of vasorelaxant agents: Synthesis and quantitative structure-activity relationship study. Chem. Pharm. Bull..

[25] Ayala A., Muñoz M.F., Argüelles S. (2014). Lipid peroxidation: Production, metabolism, and signaling mechanisms of malondialdehyde and 4-hydroxy-2-nonenal. Oxidative Med. Cell. Longev..

[26] Gupta S., Sharma S.C., Singh B. (1994). Changes in the composition and peroxidation of yeast membrane lipids during ethanol stress. Acta Microbiol. Immunol. Hung..

[27] Gazdag Z., Máté G., Certik M., Türmer K., Virág E., Pócsi I., Pesti M. (2014). tert-Butyl hydroperoxide-induced differing plasma membrane and oxidative stress processes in yeast strains BY4741 and erg5Δ. J. Basic Microbiol..

[28] Henry S.A., Kohlwein S.D., Carman G.M. (2012). Metabolism and regulation of glycerolipids in the yeast *Saccharomyces cerevisiae*. Genetics.

[29] Petranovic D., Tyo K., Vemuri G.N., Nielsen J. (2010). Prospects of yeast systems biology for human health: Integrating lipid, protein and energy metabolism. FEMS Yeast Res..

[30] Howlett N.G., Avery S.V. (1997). Induction of lipid peroxidation during heavy metal stress in *Saccharomyces cerevisiae* and influence of plasma membrane fatty acid unsaturation. Appl. Environ. Microbiol..

[31] Sattlegger E., Schiemann A.H. (2026). *Saccharomyces cerevisiae* as a model for mammalian diseases. FEMS Yeast Res..

[32] Coşkun D., Ahmedzade M. (2008). 3-(Substituted aryl)-1-(benzofuran-2-yl)-2-propenones, Part 1: Synthesis and characterization of some novel chalcones. Synth. Commun..

[33] Coşkun D., Ahmedzade M., Kirbağ S. (2011). 3-(Substituted aryl)-1-benzofuranyl-2-propenones: Antimicrobial properties of some chalcones-type compounds and their 2-pyrazoline derivatives. J. Chem..

[34] Miceli M., Roma E., Rosa P., Feroci M., Loreto M.A., Tofani D., Gasperi T. (2018). Synthesis of Benzofuran-2-One Derivatives and Evaluation of Their Antioxidant Capacity by Comparing DPPH Assay and Cyclic Voltammetry. Molecules.

[35] Lahsasni S.A., Al Korbi F.H., Aljaber N.A. (2014). Synthesis, characterization and evaluation of antioxidant activities of some novel chalcones analogues. Chem. Cent. J..

[36] Tok F., Koçyiğit-Kaymakçıoğlu B. (2020). Design, Synthesis and Biological Screening of Novel 1,5-Diphenyl-3-(4-(trifluoromethyl)phenyl)-2-pyrazoline Derivatives. Acta Chim. Slov..

[37] Ali I., Rafique R., Khan K.M., Chigurupati S., Ji X., Wadood A., Rehman A.U., Salar U., Iqbal M.S., Taha M. (2020). Potent α-amylase inhibitors and radical (DPPH and ABTS) scavengers based on benzofuran-2-yl(phenyl)methanone derivatives: Syntheses, in vitro, kinetics, and in silico studies. Bioorg. Chem..

[38] Zhu X.Q., Zhou J., Wang C.H., Li X.T., Jing S. (2011). Actual structure, thermodynamic driving force, and mechanism of benzofuranone-typical compounds as antioxidants in solution. J. Phys. Chem. B.

[39] Moalin M., van Strijdonck G.P.F., Beckers M., Hagemen G.J., Borm P.J., Bast A., Haenen G.R.M.M. (2011). A planar conformation and the hydroxyl groups in the B and C rings play a pivotal role in the antioxidant capacity of quercetin and quercetin derivatives. Molecules.

[40] Kato K., Ninomiya M., Tanaka K., Koketsu M. (2016). Effects of functional groups and sugar composition of quercetin derivatives on their radical scavenging properties. J. Nat. Prod..

[41] Ancerewicz J., Migliavacca E., Carrupt P.A., Testa B., Brée F., Zini R., Tillement J.P., Labidalle S., Guyot D., Chauvet-Monges A.M. (1998). Structure-property relationships of trimetazidine derivatives and model compounds as potential antioxidants. Free Radic. Biol. Med..

[42] La J., Kim M.J., Lee J. (2021). Evaluation of solvent effects on the DPPH reactivity for determining the antioxidant activity in oil matrix. Food Sci. Biotechnol..

[43] Bondet V., Brand-Williams W., Berset C. (1997). Kinetics and mechanisms of antioxidant activity using the DPPH• free radical method. LWT—Food Sci. Technol..

[44] Talbi W., Ghazouani T., Braconi D., Ben Abdallah R., Raboudi F., Santucci A., Fattouch S. (2019). Effects of selenium on oxidative damage and antioxidant enzymes of eukaryotic cells: Wine *Saccharomyces cerevisiae*. J. Appl. Microbiol..

[45] Piechowiak T., Balawejder M. (2019). Onion skin extract as a protective agent against oxidative stress in *Saccharomyces cerevisiae* induced by cadmium. J. Food Biochem..

[46] Sunyer-Figueres M., Mas A., Beltran G., Torija M.-J. (2021). Protective Effects of Melatonin on *Saccharomyces cerevisiae* under Ethanol Stress. Antioxidants.

[47] Janero D.R. (1990). Malondialdehyde and thiobarbituric acid-reactivity as diagnostic indices of lipid peroxidation and peroxidative tissue injury. Free Radic. Biol. Med..

[48] Gutteridge J.M.C., Halliwell B. (1990). The measurement and mechanism of lipid peroxidation in biological systems. Trends Biochem. Sci..

[49] Knight J.A., Pieper R.K., McClellan L. (1988). Specificity of the thiobarbituric acid reaction: Its use in studies of lipid peroxidation. Clin. Chem..

[50] Forman H.J., Zhang H., Rinna A. (2009). Glutathione: Overview of its protective roles, measurement, and biosynthesis. Mol. Asp. Med..

[51] Del Rio D., Stewart A.J., Pellegrini N. (2005). A review of recent studies on malondialdehyde as toxic molecule and biological marker of oxidative stress. Nutr. Metab. Cardiovasc. Dis..

[52] Jones D.P., Sies H. (2015). The redox code. Antioxid. Redox Signal..

[53] Izawa S., Inoue Y., Kimura A. (1995). Oxidative stress response in yeast: Effect of glutathione on adaptation to hydrogen peroxide stress in *Saccharomyces cerevisiae*. FEBS Lett..

[54] Sies H., Mailloux R.J., Jakob U. (2024). Fundamentals of redox regulation in biology. Nat. Rev. Mol. Cell Biol..

[55] Halliwell B., Gutteridge J.M.C. (2015). Free Radicals in Biology and Medicine.

[56] Schafer F.Q., Buettner G.R. (2001). Redox environment of the cell as viewed through the redox state of the glutathione disulfide/glutathione couple. Free Radic. Biol. Med..

[57] Thorley J. (2024). Unraveling the redox code to improve physiological research in human health and disease. Physiol. Rep..

[58] Foret M.K., Lincoln R., Do Carmo S., Cuello A.C., Cosa G. (2020). Connecting the “dots”: From free radical lipid autoxidation to cell pathology and disease. Chem. Rev..

[59] Kemp M., Go Y.-M., Jones D.P. (2008). Nonequilibrium thermodynamics of thiol/disulfide redox systems: A perspective on redox systems biology. Free Radic. Biol. Med..

[60] Gasch A.P., Spellman P.T., Kao C.M., Carmel-Harel O., Eisen M.B., Storz G., Botstein D., Brown P.O. (2000). Genomic expression programs in the response of yeast cells to environmental changes. Mol. Biol. Cell.

[61] Braconi D., Bernardini G., Santucci A. (2016). *Saccharomyces cerevisiae* as a model in ecotoxicological studies: A post-genomics perspective. J. Proteom..

[62] Hilt W., Wolf D.H. (1992). Stress-induced proteolysis in yeast. Mol. Microbiol..

[63] Dilsiz N., Celik S., Yilmaz O., Digrak M. (1997). The effects of selenium, vitamin E and their combination on the composition of fatty acids and proteins in *Saccharomyces cerevisiae*. Cell Biochem. Funct..

[64] You K.M., Rosenfield C.L., Knipple D.C. (2003). Ethanol tolerance in the yeast *Saccharomyces cerevisiae* is dependent on cellular oleic acid content. Appl. Environ. Microbiol..

[65] Sajbidor J., Ciesarova Z., Smogrovicova D. (1995). Influence of ethanol on the lipid content and fatty acid composition of *Saccharomyces cerevisiae*. Folia Microbiol..

[66] Tehlivets O., Scheuringer K., Kohlwein S.D. (2007). Fatty acid synthesis and elongation in yeast. Biochim. Biophys. Acta Mol. Cell Biol. Lipids.

[67] Klug L., Daum G. (2014). Yeast lipid metabolism at a glance. FEMS Yeast Res..

[68] Stukey J.E., McDonough V.M., Martin C.E. (1989). Isolation and characterization of OLE1, a gene affecting fatty acid desaturation from *Saccharomyces cerevisiae*. J. Biol. Chem..

[69] Pedroso N., Matias A.C., Cyrne L., Antunes F., Borges C., Malhó R., de Almeida R.F.M., Herrero E., Marinho H.S. (2009). Modulation of plasma membrane lipid profile and microdomains by H_2_O_2_ in *Saccharomyces cerevisiae*. Free Radic. Biol. Med..

[70] Rössler H., Rieck C., Delong T., Hoja U., Schweizer E. (2003). Functional differentiation and selective inactivation of multiple *Saccharomyces cerevisiae* genes involved in very-long-chain fatty acid synthesis. Mol. Genet. Genom..

[71] Komori S., Okahashi N., Iida J., Matsuda F. (2023). Lipidome variation of industrial *Saccharomyces cerevisiae* strains analyzed by LC-QTOF/MS-based untargeted lipidomics. J. Biosci. Bioeng..

[72] Shevchenko A., Simons K. (2010). Lipidomics: Coming to grips with lipid diversity. Nat. Rev. Mol. Cell Biol..

[73] Wenk M.R. (2005). The emerging field of lipidomics. Nat. Rev. Drug Discov..

[74] Yang K., Han X. (2016). Lipidomics: Techniques, applications, and outcomes related to biomedical sciences. Trends Biochem. Sci..

[75] Parks L.W., Casey W.M. (1995). Physiological implications of sterol biosynthesis in yeast. Annu. Rev. Microbiol..

[76] Abe F., Usui K., Hiraki T. (2009). Fluconazole modulates membrane rigidity, heterogeneity, and water penetration into the plasma membrane in *Saccharomyces cerevisiae*. Biochemistry.

[77] Hishmat O.H., Abd-el-Rahman A.H., Kandeel E.M., Ismail E.M. (1977). Reactions of hydroxybenzofurans. VI. Syntheses of benzofuran chalcones, hydrazones and pyrazolines of potential biological activity. Arzneimittelforschung.

[78] Ahmed M.F., Santali E.Y., El-Haggar R. (2021). Novel piperazine–chalcone hybrids and related pyrazoline analogues targeting VEGFR-2 kinase: Design, synthesis, molecular docking studies, and anticancer evaluation. J. Enzym. Inhib. Med. Chem..

[79] Ragab F.A.E.-F., Mohammed E.I., Abdel Jaleel G.A., Selim A.A.M.A.E.-R., Nissan Y.M. (2020). Synthesis of Hydroxybenzofuranyl-pyrazolyl and Hydroxyphenyl-pyrazolyl Chalcones and Their Corresponding Pyrazoline Derivatives as COX Inhibitors, Anti-inflammatory and Gastroprotective Agents. Chem. Pharm. Bull..

[80] Abe F., Hiraki T. (2009). Mechanistic role of ergosterol in membrane rigidity and cycloheximide resistance in *Saccharomyces cerevisiae*. Biochim. Biophys. Acta Biomembr..

[81] Liu J., Zhu Y., Du G., Zhou J., Chen J. (2013). Exogenous ergosterol protects *Saccharomyces cerevisiae* from D-limonene stress. J. Appl. Microbiol..

[82] Jordá T., Puig S. (2020). Regulation of Ergosterol Biosynthesis in *Saccharomyces cerevisiae*. Genes.

[83] Saha P., Yasmin A., Jha R., Passi A., Monga V., Jindal S., Goyal K. (2025). Hybrid chalcone-pyrazoline derivatives: Synthesis and in silico evaluation for anticancer potential. Future Med. Chem..

[84] Chouiter M.I., Boulebd H., Pereira D.M., Valentão P., Andrade P.B., Belfaitah A., Silva A.M.S. (2020). New chalcone-type compounds and 2-pyrazoline derivatives: Synthesis and caspase-dependent anticancer activity. Future Med. Chem..

[85] Hitge R., Smit S., Petzer A., Petzer J.P. (2020). Evaluation of nitrocatechol chalcone and pyrazoline derivatives as inhibitors of catechol-O-methyltransferase and monoamine oxidase. Bioorg. Med. Chem. Lett..

[86] Avci H., Gunduz Altintas H., Yildiz Y., Coskun D., Ari F. (2023). Effects of Chalcone Derived Compounds on Cell Cycle and Migration Capability of Human Breast and Lung Cancer Cells. Biol. Bull..

[87] Forman H.J., Davies K.J.A., Ursini F. (2014). How do nutritional antioxidants really work: Nucleophilic tone and para-hormesis versus free radical scavenging in vivo. Free Radic. Biol. Med..

[88] Kozlov A.V., Javadov S., Sommer N. (2024). Cellular ROS and antioxidants: Physiological and pathological role. Antioxidants.

[89] Sherman F. (1991). Getting started with yeast. Methods Enzymol..

[90] Treco D.A., Lundblad V. (1993). Preparation of Yeast Media. Curr. Protoc. Mol. Biol..

[91] Brand-Williams W., Cuvelier M.E., Berset C. (1995). Use of a Free Radical Method to Evaluate Antioxidant Activity. LWT—Food Sci. Technol..

[92] Ohkawa H., Ohishi N., Yagi K. (1979). Assay for lipid peroxides in animal tissues by thiobarbituric acid reaction. Anal. Biochem..

[93] Ghani M.A., Barril C., Bedgood D.R., Prenzler P.D. (2017). Measurement of antioxidant activity with the thiobarbituric acid reactive substances assay. Food Chem..

[94] Yen G.-C., Hsieh C.-L. (1998). Antioxidant activity of extracts from Du-zhong (*Eucommia ulmoides*) toward various lipid peroxidation models in vitro. J. Agric. Food Chem..

[95] Jardine D., Antolovich M., Prenzler P.D., Robards K. (2002). Liquid chromatography-mass spectrometry (LC-MS) investigation of the thiobarbituric acid reactive substances (TBARS) reaction. J. Agric. Food Chem..

[96] Mukherjee M., Nandi A., Chandra K., Saikia S.K., Jana C.K., Das N. (2020). Protein extraction from *Saccharomyces cerevisiae* at different growth phases. J. Microbiol. Methods.

[97] Lee S.J., Ramesh R., de Boor V., Gebler J.M., Silva R.C., Sattlegger E. (2017). Cost-effective and rapid lysis of *Saccharomyces cerevisiae* cells for quantitative western blot analysis of proteins, including phosphorylated eIF2α. Yeast.

[98] Ellman G.L. (1959). Tissue sulfhydryl groups. Arch. Biochem. Biophys..

[99] Sedlak J., Lindsay R.H. (1968). Estimation of total, protein-bound, and nonprotein sulfhydryl groups in tissue with Ellman’s reagent. Anal. Biochem..

[100] Lowry O.H., Rosebrough N.J., Farr A.L., Randall R.J. (1951). Protein measurement with the Folin phenol reagent. J. Biol. Chem..

[101] Vázquez J., Grillitsch K., Daum G., Mas A., Torija M.J., Beltran G. (2018). Melatonin minimizes the impact of oxidative stress induced by hydrogen peroxide in Saccharomyces and non-conventional yeast. Front. Microbiol..

[102] Phetruen T., van Dam B., Chanarat S. (2023). Andrographolide induces ROS-mediated cytotoxicity, lipid peroxidation, and compromised cell integrity in *Saccharomyces cerevisiae*. Antioxidants.

[103] Hara A., Radin N.S. (1978). Lipid extraction of tissues with a low-toxicity solvent. Anal. Biochem..

[104] Parlak A.E., Çalta M., Düşükcan M., Eroğlu M., Yılmaz Ö. (2015). The determination of fat-soluble vitamins, cholesterol content and the fatty acid compositions of shabut (Arabibarbus grypus, Heckel 1843) from Keban Dam Lake, Elazığ, Turkey. J. Fish. Sci..

[105] Emre İ., Kursat M. (2022). Biochemical parameters and antioxidant property of three *Salvia* L. taxa endemic in Turkey. Braz. J. Biol..

[106] Eder K. (1995). Gas chromatographic analysis of fatty acid methyl esters. J. Chromatogr. B Biomed. Appl..

[107] Katsanidis E., Addis P.B. (1999). Novel HPLC analysis of tocopherols, tocotrienols, and cholesterol in tissue. Free Radic. Biol. Med..

[108] López-Cervantes J., Sánchez-Machado D.I., Ríos-Vázquez N.J. (2006). High-performance liquid chromatography method for the simultaneous quantification of retinol, α-tocopherol, and cholesterol in shrimp waste hydrolysate. J. Chromatogr. A.

