# Supplementary material for: Effects of Benzofuran–Chalcone, Benzofuran–Pyrazoline, and Bis-Pyrazoline Derivatives on Redox-Related Biomarkers and Fatty Acid, Vitamin, and Sterol Profiles in Saccharomyces cerevisiae
Source: Molecules. 2026 Oct 20;31(20):3769. doi: 10.3390/molecules31203769 (PMC13707640; doi:10.3390/molecules31203769)
Supplement: Supplementary file 1 [file molecules-31-03769-s001.zip › molecules-4526692-supplementary.pdf]

Table S1. Raw DPPH absorbance values, group means, and normalized radical scavenging activities (control = 1.0 for each concentration).

Group	Concentra	Replicate 1	Replicate 2	Replicate 3	Replicate 4	Replicate 5	Average	Normalize
Control	10	0,791	0,804	0,81	0,798	0,794	0,799	1,000
Control	20	0,301	0,297	0,312	0,299	0,308	0,303	1,000
Control	30	0,12	0,131	0,129	0,123	0,137	0,128	1,000
Quercetin	10	0,6684	0,6872	0,6731	0,6905	0,6783	0,6795	0,850
Quercetin	20	0,1822	0,1936	0,1874	0,1908	0,1865	0,1881	0,620
Quercetin	30	0,0876	0,0941	0,0902	0,0925	0,0901	0,0909	0,710
1a	10	0,1927	0,2048	0,1983	0,2022	0,2015	0,1999	0,250
1a	20	0,0821	0,0872	0,0844	0,0863	0,085	0,085	0,280
1a	30	0,0361	0,0396	0,0388	0,0377	0,0398	0,0384	0,300
1b	10	0,0762	0,0831	0,0791	0,0812	0,0799	0,0799	0,100
1b	20	0,0348	0,0377	0,0359	0,0369	0,0367	0,0364	0,120
1b	30	0,0191	0,0217	0,0203	0,021	0,0204	0,0205	0,160
2a	10	0,5482	0,5671	0,5543	0,5628	0,5656	0,5596	0,700
2a	20	0,2512	0,2641	0,2566	0,2603	0,2573	0,2579	0,850
2a	30	0,0988	0,1061	0,1017	0,1035	0,1019	0,1024	0,800
2b	10	0,2721	0,2864	0,2785	0,2822	0,2798	0,2798	0,350
2b	20	0,2056	0,2187	0,2109	0,2142	0,2126	0,2124	0,700
2b	30	0,0741	0,0793	0,0762	0,0781	0,0763	0,0768	0,600
2c	10	0,2321	0,2464	0,2386	0,2419	0,24	0,2398	0,300
2c	20	0,0933	0,1006	0,0964	0,0982	0,097	0,0971	0,320
2c	30	0,0429	0,0466	0,0441	0,0455	0,0449	0,0448	0,350

Table S2. Raw TBARS absorbance values, group means, and normalized MDA values (control = 1.0).

Group	Concentra	Replicate 1	Replicate 2	Replicate 3	Replicate 4	Replicate 5	Average	Normalize
Control	10	0,082	0,086	0,091	0,088	0,088	0,087	1,000
Control	50	0,161	0,176	0,181	0,17	0,172	0,172	1,000
Control	100	0,022	0,027	0,031	0,026	0,034	0,028	1,000
FR	10	0,08	0,085	0,094	0,086	0,09	0,087	1,000
FR	50	0,238	0,249	0,266	0,274	0,258	0,257	1,494
FR	100	0,042	0,045	0,048	0,051	0,049	0,047	1,679
Resver.	10	0,084	0,088	0,086	0,091	0,086	0,087	1,000
Resver.	50	0,194	0,201	0,209	0,214	0,212	0,206	1,198
Resver.	100	0,0338	0,0352	0,0367	0,038	0,0368	0,0361	1,289
1a	10	0,13	0,136	0,145	0,141	0,143	0,139	1,598
1a	50	0,276	0,287	0,294	0,301	0,302	0,292	1,698
1a	100	0,026	0,031	0,034	0,037	0,037	0,033	1,179
1b	10	0,06	0,066	0,07	0,073	0,076	0,069	0,793
1b	50	0,262	0,271	0,278	0,286	0,278	0,275	1,599
1b	100	0,045	0,047	0,051	0,055	0,052	0,05	1,786
2a	10	0,121	0,127	0,131	0,138	0,133	0,13	1,494
2a	50	0,21	0,218	0,225	0,232	0,23	0,223	1,297
2a	100	0,043	0,046	0,048	0,051	0,047	0,047	1,679
2b	10	0,114	0,119	0,122	0,128	0,122	0,121	1,391
2b	50	0,198	0,203	0,208	0,211	0,21	0,206	1,198
2b	100	0,039	0,042	0,045	0,048	0,046	0,044	1,571
2c	10	0,127	0,136	0,141	0,146	0,145	0,139	1,598
2c	50	0,281	0,286	0,293	0,3	0,3	0,292	1,698
2c	100	0,043	0,049	0,052	0,055	0,051	0,05	1,786
